# Development of the ‘COuld it Be RA’ (COBRA) tool to facilitate early identification of people at risk of developing rheumatoid arthritis in primary care

**DOI:** 10.1136/rmdopen-2025-005957

**Published:** 2026-02-15

**Authors:** Heidi J Siddle, Anna M Anderson, Elizabeth M A Hensor, Kulveer Mankia, Paul Emery, Suzanne H Richards

**Affiliations:** 1 Leeds Institute of Rheumatic and Musculoskeletal Medicine, University of Leeds, Leeds, UK; 2 Podiatry Department, Leeds Teaching Hospitals NHS Trust, Leeds, UK; 3 Leeds Institute of Health Sciences, University of Leeds, Leeds, UK; 4 NIHR HealthTech Research Centre in Accelerated Surgical Care, Leeds, UK; 5 NIHR Leeds Biomedical Research Unit, Leeds, UK

**Keywords:** Anti-Citrullinated Protein Antibodies, Arthritis, Rheumatoid, Risk Factors, Qualitative research

## Abstract

**Objectives:**

We aimed to develop a new complex intervention, the ‘COuld it Be RA’ (COBRA) tool, to support the implementation of a clinical prediction model to identify people likely to be anti-cyclic citrullinated peptide (CCP) positive and at risk of rheumatoid arthritis in primary care.

**Methods:**

The COBRA tool was developed using the UK Medical Research Council and National Institute for Health and Care Research complex intervention research framework. This study involved three sequential phases with primary care clinicians: a qualitative descriptive study, clinician consultation engagement workshops and a think-aloud interview study. Ethical approval was obtained for all three phases.

**Results:**

Sixteen primary care clinicians participated in semistructured interviews to identify barriers and facilitators. An initial list of nine candidate components for the intervention, including design considerations, was developed. During phase 2 workshops with eight participants, four components were prioritised as ‘Must have’ or ‘Should have’: the clinical decision support system (CDSS); guidance on using the CDSS/associated actions; evidence for the CDSS; patient education resources. A COBRA tool prototype incorporating these components was developed.

Twelve participants tested the prototype during think-aloud interviews. Key perceived benefits of the COBRA tool included supporting clinicians’ decision-making and reducing unnecessary anti-CCP testing. Over 40 changes were made to the COBRA tool.

**Conclusion:**

Our research included the views of clinicians and PPI representatives and was underpinned by a complex intervention research framework. This was critical to understanding barriers and facilitators to implementing the clinical prediction model in primary care and developing the COBRA tool.

**WHAT IS ALREADY KNOWN ON THIS TOPIC**

- Early identification and initiation of treatment of rheumatoid arthritis (RA) improves long-term outcomes; identification of individuals at risk of RA may delay or prevent the onset of RA.

**WHAT THIS STUDY ADDS**

- This study describes the development of the COuld it Be RA tool, a simple clinical decision support system to facilitate early identification of people at risk of developing RA in primary care.

**HOW THIS STUDY MIGHT AFFECT RESEARCH, PRACTICE OR POLICY**

- Identification of individuals at risk of RA in primary care will prompt earlier referral to rheumatology services for monitoring, diagnosis and treatment, including prevention initiatives.

## Introduction

 Rheumatoid arthritis (RA) develops in stages; systemic autoimmunity and other pathological processes occur well before the onset of clinically detectable arthritis, which is when a diagnosis of RA is made.^1^ Increasing evidence from clinical trials suggests that intervention in the pre-arthritis phase (ie, in ‘at-risk’ patients) could reduce the likelihood of RA developing, delay the onset of RA or reduce the severity of future RA should it develop.^2^,^5^

People with musculoskeletal (MSK) problems, including those with RA, typically first present in primary care.^6^ For people with RA, there is evidence that delays occur between the onset of symptoms and booking an appointment to seek help from their GP, and the time spent in primary care before being referred to a specialist, both contribute to the total delay between patients developing symptoms and being assessed by a rheumatologist.^7^,^9^ General practitioners (GPs) have reported finding it particularly challenging to identify the earliest phases of RA, as initial symptoms can be non-specific and are similar to those of many other conditions.^10 11^ As well as delaying diagnosis and treatment, referral delays in primary care can cause frustration and anxiety for patients and negatively affect their relationships with GPs.^12^ Furthermore, there appears to be variation in how GPs use and interpret blood tests when they suspect a patient has RA. These issues all contribute to referral delays.^10 11^

In this pre-arthritis phase of RA, it is well established that at-risk individuals develop circulating anticitrullinated protein antibodies (ACPA) before developing joint swelling (synovitis).^1^ Targeted anti-cyclic citrullinated peptide (CCP) testing (for ACPA) provides an opportunity to identify people with MSK symptoms who are ‘at risk of developing RA’ before developing synovitis (clinically detectable arthritis). These individuals may then be promptly referred to rheumatology specialist services for monitoring, early diagnosis and rapid initiation of treatment.

We have developed a prediction model to identify patients presenting to primary care with new-onset MSK symptoms without synovitis who are likely to be anti-CCP positive and therefore at risk of developing RA.^13^ However, implementing prediction models in an overstretched primary care setting can be challenging; clinicians describe only using them in consultations when the models are perceived to be useful (eg, readily accessible and aiding in clinical decision-making), and/or they assist in meeting best-practice guidance or financial/performance targets.^14^ The solution lies in a simple tool, a clinical decision support system (CDSS)^15^ requiring organisational, technology and behaviour change to support the implementation in practice.^16^ This study describes the development of a new complex intervention, the ‘COuld it Be RA’ (COBRA) tool to support primary care clinicians to identify people likely to be anti-CCP positive and at risk of RA.

## Methods

### Overall design

**Figure 1:**
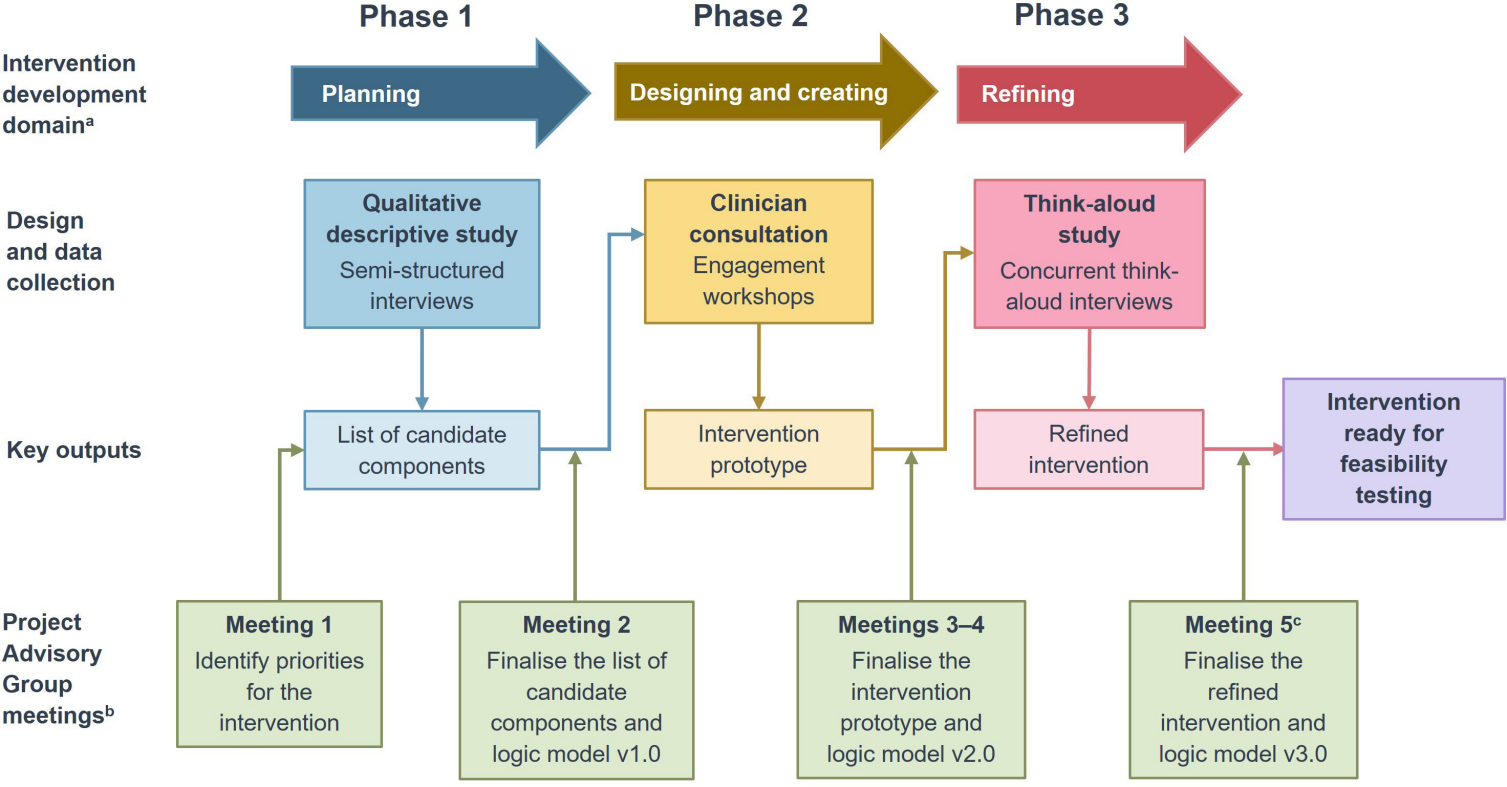
Project overview. Overview of the project phases and Project Advisory Group involvement. Online supplemental appendix S5 provides an image description. ^a^Intervention development domains based on O’Cathain *et al*.^19^ ^b^Only Project Advisory Group actions that are directly focused on developing the intervention and logic model are shown. ^c^Meeting 5 was held while the Phase 3 interviews and intervention refinement were ongoing, so follow-up was undertaken via email.

The study design was underpinned by the Medical Research Council/National Institute for Health and Care Research complex intervention framework^17^ and associated resources,^18 19^ and drew on practical guidance from the Behaviour Change Wheel (BCW)^20^ and person-based approach.^21^ This study involved three sequential phases (figure 1): a qualitative descriptive study, clinician consultation engagement workshops and a think-aloud interview study. A process-oriented logic model was developed using a staged approach, representing the intervention’s programme theory, that is, how it is anticipated to work.^22^ The reporting was informed by the GUIDance for the rEporting of intervention Development checklist.^23^

### Project advisory group

The study was overseen by a project advisory group (PAG) involving patient and public involvement (PPI) representatives, primary care clinicians, rheumatologists and researchers. The full PAG met five times throughout the project (figure 1). Additional meetings/correspondence were arranged with individual PAG members as needed. The PAG was involved in activities such as monitoring the study progress, offering advice on the study conduct and helping to develop the intervention.

### Patient and public involvement

The study grant application was informed by a PPI stakeholder group. Two PPI group members subsequently reviewed a summary of the study protocol and did not suggest any changes. Five PPI representatives joined the PAG over the course of the study, with approximately two PPI representatives actively contributing at any one time. In addition to their main PAG roles, two PAG PPI representatives helped develop a plain English summary and one copresented a public engagement video.

### The anti-CCP prediction model

The anti-CCP prediction model, forming the basis of the COBRA tool, was initially developed as a score chart (table 1)^13^ for completion with patients presenting to primary care with new-onset MSK symptoms without synovitis. The scores are summated to obtain a total score, with individuals referred for anti-CCP testing if the total score met the score threshold. 8-point and 11-point thresholds were considered, which would lead to anti-CCP testing of approximately 40% or 19% of patients with new-onset MSK symptoms, respectively^13^ (online supplemental table S1).

**Table 1:** Anti-CCP prediction model

Predictor variables	Score
Joint pain: back	–3
Joint pain: neck	–2
Joint pain: knees	–1
Joint pain: wrists	4
Sex: male	3
First degree relative* with RA	3
Joint pain: feet/toes	3
Joint pain: hands/fingers	3
Joint pain: shoulders	3
Smoking status: ever	2
Joint pain: thumbs	1
**Total score**	

Scores to be added up to obtain one total score, on a scale of –6 to 22 (for bilateral joints, pain in one or both sides should be considered an affirmative response).

Table reproduced without modifications from table 2 in Siddle *et al*^13^ under the terms of the CC BY-NC 4.0 licence (https://creativecommons.org/licenses/by-nc/4.0/).

* Mother, father, brother, sister, son or daughter.

CCP, cyclic citrullinated peptide; RA, rheumatoid arthritis.

Box 1 **Target behaviours related to implementing the anti-CCP prediction model in primary care**

- Using a CDSS incorporating the anti-CCP prediction model with appropriate patients.
- Organising for patients to have an anti-CCP test when supported by the CDSS.
- Referring patients with a positive anti-CCP test to rheumatology services.
- Having appropriate discussions about anything related to RA, anti-CCP testing or rheumatology referrals with patients.

Reproduced without modification from Box 1[Boxed-text B1] in Anderson *et al*^30^ under the terms of the CC BY licence (https://creativecommons.org/licenses/by/4.0/).Anti-CCP, anti-cyclic citrullinated peptide; CDSS, clinical decision support system; RA, rheumatoid arthritis.

### Participant selection and recruitment

Practising primary care clinicians (excluding trainees) in England were eligible if they were undertaking regular weekly clinics for a provider of NHS primary care services, which included assessing people presenting with new-onset MSK symptoms without synovitis. GPs and first contact practitioners (FCPs) were included in all phases. Advanced nurse practitioners (ANPs) were also included in phase 3. Participating in a previous phase was not an exclusion criterion.

To ensure diverse perspectives were considered, maximum variation purposive sampling was employed based on years of experience, organisation type(s) and anti-CCP requesting practices (all phases), and experience of recruiting participants to a previous primary care anti-CCP testing study^12^ (phases 1 and 2 only). Participants were recruited via professional networks, clinical providers and snowballing. In line with the qualitative design, sample size ranges of 10–20, 8–14 and 6–12 for phase 1, 2 and 3, respectively, rather than exact sample sizes, were prespecified (online supplemental table S2).^24^,^29^

### Data collection and analysis

All participants self-reported data about their professional characteristics during the screening process and provided electronic consent. The interviews and workshops were recorded, transcribed verbatim and pseudo-anonymised, then verified by AMA. Field notes were recorded during/after each interview/workshop. Data were managed using NVivo software (Release V.1.6, then NVivo V.14), Microsoft Excel and Microsoft Word.

#### Phase 1: identifying intervention components

Semistructured interviews were conducted to explore how primary care clinicians currently identify and refer patients with suspected RA, and the behaviours required to implement the anti-CCP prediction model in primary care. Details of the phase 1 data collection and framework analysis are reported elsewhere.^30^ This paper reports the behavioural analyses used to identify candidate components for the intervention.

Applying the BCW approach, we identified and mapped target behaviours relating to implementing the anti-CCP prediction model in primary care (Box 1).

AMA and HJS created a behavioural analysis table for each target behaviour by mapping barriers and facilitators identified through the framework analysis to components of the Capability Opportunity Motivation model of behaviour (COM-B), BCW intervention functions, behaviour change techniques from the behaviour change technique taxonomy (v1) and candidate intervention components. The tables were refined based on feedback from the wider study team. Intervention priorities identified by the PAG (online supplemental appendix S1) were considered throughout this process to ensure the candidate intervention components were likely to be feasible and acceptable. The candidate intervention components from all the tables were collated for Phase 2.

#### Phase 2: prioritising intervention components and prototype development

Two 90 min engagement workshops were conducted via videoconferencing to refine and prioritise the candidate intervention components identified in phase 1. AMA and HSJ co-facilitated both workshops using a topic guide (online supplemental appendix S2). EMAH attended the second workshop to respond to statistical queries regarding the prediction tool. Participants were provided with an overview of the study, workshop purpose, candidate components and workshop format (table 2) before attending.

**Table 2:** Workshop format

Item	Summary
Welcome and introduction	Welcome and introduction to the workshop, including a brief presentation covering the workshop agenda, project background and candidate components.
Activity 1: additional components	Identification and discussion of any additional components that participants felt should be included in the intervention.
Activity 2: prioritisation round 1	Online polls to prioritise candidate components using the MoSCoW model.^39^ ^40^
Activity 3: candidate components discussion	Discussion of candidate components rated as ‘Must have’ or ‘Should have’ including considerations for including the component in the intervention based on the APEASE^20^ criteria, and design considerations for the component.
Activity 4: combining, implementing and embedding discussion	Discussion of how the intervention components should be combined, implemented and embedded into routine practice in primary care.
Activity 5: prioritisation round 2	Online polls to prioritise the pre-specified and additional candidate intervention components using the MoSCoW model.^39 40^
Workshop closure	Workshop closure and thanking of participants.

APEASE, affordability, practicability, effectiveness and cost-effectiveness, acceptability, side-effects/safety and equity; MoSCoW model, Must have, Should have, Could have, Would like model.

Data from both prioritisation rounds (activities 2 and 5) were analysed using descriptive statistics. Each component was assigned a priority level using the MoScoW (Must have, Should have, Could have, Would like) model based on participant voting. Achievement of consensus was defined as ≥75% of participants selecting the same rating as that is a commonly used approach in consensus development studies.^31^

Qualitative data were analysed using deductive content analysis.^32^ AMA and HJS coded data and then created a refined and prioritised list of intervention components based on the categorisation matrix, round 2 priority ratings of all participants considered together, and research team and PAG discussions. The research team used this list of components and PAG input to draft the intervention content and commissioned a web design/development company, ‘Frank Design Limited’^33^, to create an intervention prototype as a standalone website. A demo version was created initially and refined based on feedback from the research team and PAG members. A live version of the prototype was taken forward to Phase 3.

#### Phase 3: user testing to refine the prototype

Concurrent think-aloud interviews via video-conferencing were conducted to evaluate the usability of the prototype and explore clinicians’ perspectives of it. Fictional clinical vignettes, designed to fully test different presentations, were used to support participants to work through the prototype. AMA conducted the interviews using a topic guide (online supplemental appendix S3).

An interactive think-aloud interview style was employed to enable participants’ experiences and perspectives of using the prototype to be explored in depth.^34 35^ Each participant worked through the prototype while saying everything they were thinking out loud, with the interviewer prompting/guiding where appropriate to specific content. After they had viewed the main prototype pages, the participant was asked to use the prototype with any of the vignettes for whom they felt it was clinically indicated (online supplemental table S3). The participant was then asked brief semistructured questions to explore their overall views of the prototype.

Data collection and analysis were conducted concurrently to enable the prototype to be iteratively refined. A rapid analysis method developed for the refinement phase of person-based intervention development studies was employed.^28^ AMA and HJS worked through transcripts to identify positive and negative comments about the prototype, discuss potential changes that could address the negative comments and prioritise each change using bespoke criteria (table 3). AMA, HJS and SHR decided which changes to implement. Changes were implemented on an ongoing basis to enable subsequent interviews to be used to help determine whether the changes made were useful. Summaries of all the comments and changes were recorded in a ‘table of changes’.^36^

**Table 3:** Criteria for implementing changes to the prototype

Change code*	Reason for change criteria*	Importance level	Time consuming to implement†	Priority‡
FSR	Important for the intervention functioning/navigation, safety or compliance with relevant regulations/guidelines.	1	No	Must have
			Yes	Must have
PAG	Consistent with the views of the Project Advisory Group.	2	No	Should have
LIT	Consistent with existing literature.		Yes	Could have
BEH (target behaviour)	Likely to impact engagement with one or more of the COBRA tool’s target behaviours (Box 1)§ This includes changes that may directly impact the behaviours and/or those that may impact precursors to the behaviours, eg, acceptability, accessibility, persuasiveness.			
REP	Addresses a point repeated by more than one participant.	3	No	Could have
EAS	Easy and uncontroversial to implement as it does not require any substantial design changes, eg, amending the text to improve clarity and/or readability.		Yes	Would like
NNA (reason)	Not needed or appropriate, eg, due to not meeting any of the criteria listed above and/or contradicting one or more of the criteria listed above.	N/A	N/A	N/A

* Change code and reasons for change criteria adapted from Bradbury *et al*,^28^ Anderson *et al*,^41^ and a person-based approach table of changes template.^42^

† Changes were classed as time-consuming to implement if they required substantial programming time and/or the development of a new page or resource such as video or PDF document.^41^

‡ If a change was supported by more than one reason, the priority was based on the reason with the highest importance level.

§ Target behaviour 3 was updated to ‘Seek rheumatology guidance for patients with a positive anti-CCP test’. The other three target behaviours were as specified for phase 1.

BEH, behaviours; CCP, cyclic citrullinated peptide; EAS, Easy; FSR, functioning/navigation, safety or compliance with relevant regulations/guidelines; LIT, literature; NNA, not needed or appropriate; PAG, project advisory group; REP, repeated.

## Results

### Phase 1: identifying intervention components

Eight GPs and eight FCPs participated in semistructured interviews.^30^ The barriers and facilitators mapped to all the COM-B components except for physical capability (table 4). Online supplemental tables S4–S7 present the full behavioural analyses.

**Table 4:** Behavioural analyses summary

Target behaviour	COM-B^43^ component	Barrier (B) or facilitator (F) example
Using a CDSS incorporating the anti-CCP prediction model with appropriate patients.	Psychological capability	Uncertainty about which patients to use the CDSS with (B)
	Physical opportunity	Accessing the CDSS being quick and easy (F)
	Social opportunity	Concerns about GPs perceiving FCPs are requesting too many anti-CCP tests (B)
	Reflective motivation	Concerns about cost implications of using the CDSS (B)
	Automatic motivation	Having prompts to use the CDSS (F)
Organising for patients to have an anti-CCP test when supported by the CDSS.	Psychological capability	Lack of awareness of the anti-CCP test on the blood test requesting system (B)
	Physical opportunity	Being able to directly request anti-CCP tests (F)
	Social opportunity	GPs not trusting FCPs to act autonomously (B)
Referring patients with a positive anti-CCP test to rheumatology services.	Psychological capability	Being unsure about some rheumatology referral procedures (B)
	Physical opportunity	Being able to directly refer patients to rheumatology services (F)
	Social opportunity	Potential lack of acceptance of referrals by the rheumatology team (B)
	Reflective motivation	Concerns about inappropriately burdening rheumatology services and increasing waiting times (B)
Having appropriate discussions about anything related to RA, anti-CCP testing or rheumatology referrals with patients.	Psychological capability	Having the knowledge and skills to involve patients in decision making (F)
	Physical opportunity	Limited time to have discussions with patients (B)
	Social opportunity	Perceiving it is normal practice not to involve patients in decisions about whether to have blood tests (B)
	Reflective motivation	Believing discussions will be helpful and/or reassuring for patients (F)

Anti-CCP, anti-cyclic citrullinated peptide; CDSS, clinical decision support system; COM-B, Capability Opportunity Motivation model of behaviour; FCP, first contact practitioner; GP, general practitioner; RA, rheumatoid arthritis.

The behavioural analysis tables were used to develop an initial list of nine candidate components for the intervention, including design considerations for each component (table 5, online supplemental table S8).

**Table 5:** Initial list of candidate components

Candidate component	Design consideration example
CDSS	Provide the CDSS in more than one format (eg, website, embedded in electronic health records).
Evidence supporting use of the CDSS	Provide evidence about how the anti-CCP model was developed.
Guidance on using the CDSS and undertaking the associated behaviours	Provide brief guidance on using the CDSS and follow-up actions that covers which patients to use/not use the CDSS with.
Information covering key points about the CDSS	Provide information that highlights potential benefits of using the CDSS, including for supporting clinical reasoning and improving patient outcomes.
Video of and/or quotes from MDT professionals supporting use of the CDSS and associated behaviours	Include rheumatologists reinforcing that referring patients with a positive anti-CCP test is appropriate and unlikely to overburden their services or increase waiting times.
Reminders about using the CDSS	Provide reminders in formats such as email reminders and pop-up reminders.
Summary information about the CDSS for sharing via appropriate routes	Include brief information about the purpose of the CDSS and its potential benefits.
Clinical audit tool	Support clinicians to record their use of the CDSS and if/how it affected their practice.
Patient education resources	Provide easily accessible patient education resources such as a leaflet that can be shared via text.

Anti-CCP, anti-cyclic citrullinated peptide; CDSS, clinical decision support system; MDT, multidisciplinary.

Two key refinements to the proposed CDSS were made based on the phase 1 findings. First, the 11-point threshold was selected (online supplemental table S1) as multiple participants felt that anti-CCP testing of 19% rather than 40% of patients with new-onset MSK symptoms would be more appropriate from a workload perspective.

Second, the variables were ordered as first-degree relative with RA, sex, smoking history and the joint pain variables from head to toe as several participants felt that would be preferable to the numerical ordering. Additionally, one participant highlighted that having a first degree relative first would be helpful:

I think if I was putting them in a table, I'd put the first degree relative first because that’s the one that we forget to ask about. And sometimes if we don't always complete everything in the consultation, we'll go back and do it afterwards. But if we've forgotten to ask about something, then it’s a complete pain in the neck. (GP1-8)

### Phase 2: prioritising intervention components and prototype development

Eight individuals participated in the two workshops (three GPs, five FCPs). One participant has previously taken part in phase 1. Online supplemental table S9 provides the participants’ characteristics.

No additional intervention components were suggested. Based on the overall round 2 priority ratings, two components were rated as ‘Must have’ (CDSS and guidance on using the CDSS and undertaking the associated behaviours), two as ‘Should have’ (evidence supporting the use of the CDSS and patient education resources) and five as ‘Could have’ (online supplemental table S10).

**Figure 2:**
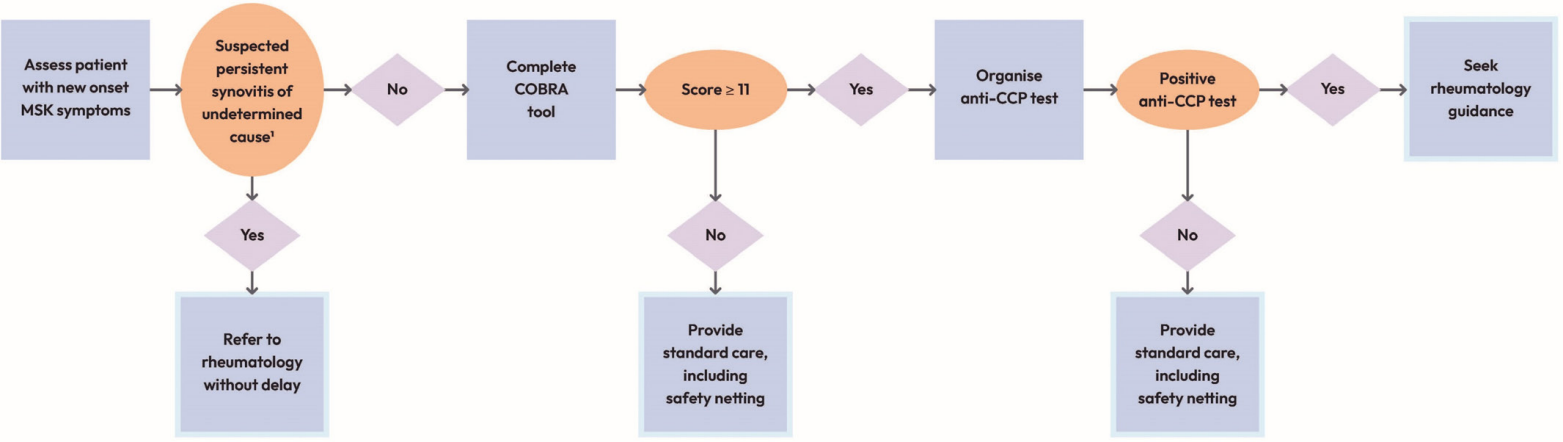
The COBRA tool flowchart. Primary care clinicians are provided with the following information under ‘Patient pathways’ in the COBRA tool website to be used with the COBRA tool flow chart: patient with soft tissue joint swelling (synovitis: refer to rheumatology without delay as advised in the National Institute for Health and Care Excellence (NICE) guideline for RA [NG100]. Patient with new-onset MSK symptoms without synovitis: complete the COBRA tool and follow the steps in the flowchart below. Even if the patient has pain in only one joint, please complete the COBRA tool. When the anti-CCP test is not recommended or the result is negative, provide standard care. This should include safety netting as the patient may still develop RA or have another type of inflammatory arthritis. An expandable box to view a text version of the flowchart is available on the COBRA tool website and is provided in online supplemental appendix S6. ¹See the National Institute for Health and Care Excellence (NICE) guideline for rheumatoid arthritis [NG100]. Anti-CCP, anti-cyclic citrullinated peptide; COBRA tool, Could it be RA? tool; MSK, musculoskeletal.

All the ‘Must have’ and ‘Should have’ components and one ‘Could have’ component (information covering key points about the CDSS) were included in the refined and prioritised list of intervention components (online supplemental table S11). The ‘Could have’ component was selected for inclusion by the research team because it links closely with the other components. The design considerations for the five included components were refined by removing, amending and adding considerations. For example, in the initial list of candidate components (online supplemental table S8), four potential formats for delivering guidance were listed: brief text, a flowchart, a video and case studies. Only a flowchart (figure 2) was retained in the refined and prioritised list of components (online supplemental table S11), as the phase 2 participants felt that would be quickest and easiest to follow.

Nearly all the design considerations included in the refined and prioritised list of components were addressed when developing the prototype intervention. The main exception was related to the CDSS delivery format. As with phase 1, the phase 2 findings suggested it would be helpful to provide the CDSS in a variety of formats. Although none of the phase 2 participants stated they would like a paper-based version, other suggestions included providing a patient-completed questionnaire, an electronic format integrated within electronic health record systems and a standalone website:

Or I think having it, sort of, embedded in the clinical system is definitely a good idea, that you can fill it in, it populates in the notes, it’s there as a record, it’s clear, it’s in the score, but also maybe having, you know, on a separate, standalone website, I think would be beneficial as well. (FCP2-11)

While providing the CDSS in more than one format was included as a key design consideration (online supplemental table S11), the research team and PAG agreed that developing the CDSS as a standalone website only would be most feasible for this study, with the aim of adapting the refined version to other delivery formats (eg, embedded within electronic systems) in the future. Based on discussions with the PAG, the standalone website was called the COBRA tool, as ensuring the intervention has a memorable and relatively unique name was identified as important:

Often, you have to think about clever words that will allow it to pop up, so if you were to label the decision tool the Anti-CCP Decision Tool, I would never find it on Google. So just thinking about how searchable it is […] (GP2-10)

The COBRA tool prototype taken forward to phase 3 included a homepage, three main pages and four supporting pages (table 6). Figure 2 provides the COBRA tool flowchart.

**Table 6:** COBRA tool prototype summary

Page	Summary	Key features
Welcome to the COuld it Be RA? (COBRA) tool	Homepage providing the CDSS and accompanying information and guidance	Banner with text explaining who the intended users of the CDSS are and which patients to use it with; the funder logo, acknowledgement and disclaimer; and ‘Jump to’ buttons for the CDSS and patient pathways sections. Brief text summarising the CDSS purpose and potential benefits. Two expandable boxes with guidance on how to use the tool. CDSS presented as a form with radio buttons for each variable. ‘Submit’ button for generating the CDSS tool output, which includes whether the anti-CCP test is recommended based on the score obtained, a hyperlink to the ‘Clinical discussion points’ page, key points related to interpreting the output, and a summary of the output to copy, print or save. ‘Patient pathways’ text and flow chart summarising the suggested pathway for patients with synovitis and patients with new-onset MSK symptoms without synovitis. Button to view the flow chart in full screen mode. Expandable box to view a text version of the flow chart.
COBRA tool evidence	Main page with evidence related to RA and the COBRA tool	Brief text summarising how the COBRA tool was developed. Four expandable boxes summarising anti-CCP prediction model properties. Brief text summarising the COBRA tool development with two expandable boxes listing the research team members’ names and Project Advisory Group members’ names. Six expandable boxes summarising the COBRA tool rationale. Seven expandable boxes with hyperlinks to key papers related to the COBRA tool.
Clinical discussion points	Main page summarising key points to discuss with patients	Brief text highlighting the potential benefits of discussing points about anti-CCP testing with patients and the importance of providing tailored advice. Four expandable boxes with key points to discuss with patients at different stages of the care pathway, including hyperlinks to relevant webpages, eg, for healthy lifestyle support (all boxes), and a hyperlink to the ‘Patient resources’ page and a button to download the COBRA tool patient booklet (‘Explaining a positive anti-CCP test result’ box).
Patient resources	Main page with resources intended for patients who are anti-CCP positive	Button to download the COBRA tool patient booklet. Brief text signposting to the ‘Help’ page and summarising key information about the anti-CCP test. Four expandable boxes with further information about RA and the anti-CCP test, with hyperlinks to further information about RA. An alert box and expandable box with guidance on what patients should do if their symptoms change. Placeholders for photographs showing swelling at different joints. Brief text and expandable boxes with information on lifestyle-related factors that may influence the risk of RA, including hyperlinks to relevant webpages. Brief text summarising the implications of a negative anti-CCP test result.
Supporting pages	Pages required for regulatory and accessibility purposes	‘Help’ page with brief text on how to address problems with using the COBRA tool website and five expandable boxes with guidance on using the website. ‘Contact us’ page with a contact form for questions about the website. ‘Accessibility statement’ with the accessibility statement for the COBRA tool. ‘Privacy and cookies’ policy page with the privacy and cookies policy for the COBRA tool.
All pages	N/A	Header with the COBRA tool, Leeds Teaching Hospitals NHS Trust and University of Leeds logos. ‘Log out’ button*, search box, and dropdown menu. Box links to the three main pages (or two main pages and the ‘Help’ page if the user is viewing one of the main pages). Footer with links to the ‘Contact us’, ‘Privacy and cookies policy’ and ‘Accessibility statement’ pages and the University of Leeds Terms of use. Accessibility toolbar that allows users to change the language, text size and colour contrast.

* Necessary for the prototype and testing but would not be required for use in clinical practice.

Anti-CCP, anti-cyclic citrullinated peptide; CDSS, clinical decision support system; COBRA, COuld it Be RA?; MSK, musculoskeletal; NHS, National Health Service; RA, rheumatoid arthritis.

### Phase 3: user testing to refine the prototype

Twelve participants (seven GPs, three FCPs, two ANPs) participated in a think-aloud interview (duration 41–64 min; median 59 min). Online supplemental table S12 provides the participants’ characteristics.

Most participants had a positive overall view of the COBRA tool, reporting it is clear, helpful and easy to use:

So, I do think it’s really, a really, sort of, patient-friendly, clinician-friendly tool. There’s nothing intricate on there that’s going to complicate things or make you feel like you need to, sort of, ask more in-depth, you know, it’s quite to the point. (ANP3-2)

Key perceived benefits of the COBRA tool included supporting clinicians’ decision-making and reducing unnecessary use of the anti-CCP test. This was considered particularly helpful by participants who viewed the current approach to anti-CCP testing as inconsistent or reported they tend to *just tag on* (GP3-12) the anti-CCP test. Multiple participants also felt that using the COBRA tool could support discussions with patients:

But this just adds more weight to that discussion with the patient about why we’re not going to do further tests. (GP3-13)

One GP had a strongly negative view of the COBRA tool and stated he would not use it in practice. This participant was concerned about the overall concept of using the anti-CCP test to identify people at risk of RA and how at-risk patients would be managed:

I think the crucial thing is, I don’t want a tool for something, to find something early that I can't make a good difference to. (GP3-17)

Other participants also raised concerns about specific aspects of the COBRA tool, such as certain text appearing unclear or confusing. To help address the issues identified, over 40 changes were made to the COBRA tool. The most notable changes were intended to address the issue of participants potentially using the COBRA tool with patients with suspected RA/synovitis:

I think if I had a patient where I was suspecting rheumatoid, I’d go and do that for completeness or just for that reassurance value. (FCP3-14)

This was considered a potential safety issue as, if clinicians use the tool with patients with synovitis and it suggests the anti-CCP test is not needed, it may give clinicians false reassurance that the anti-CCP test and a rheumatology referral are not required. As a consequence, a red alert banner was added directly above the CDSS (online supplemental appendix S4). Notwithstanding this change, five subsequent participants still indicated they might use the tool with patients with suspected synovitis/RA. Following discussions with the PAG and the tenth interview, a screening question was added above the CDSS (online supplemental appendix S4).

Another notable issue was that all participants indicated they would not use the COBRA tool with certain patients, such as those with pain in only one joint. The ‘patient pathways’ text was amended to clarify this, but subsequent participants still indicated they would probably only use the COBRA tool with patients with specific characteristics or symptoms (eg, small joint pain), or where there was diagnostic uncertainty. Participants generally felt that using the tool with all patients with new-onset MSK symptoms without synovitis would not be appropriate or feasible:

I just think that, yeah, like, the definition of new-onset MSK pain, I think that’s just a little bit too broad in my thinking because I see so many MSK patients, I just don’t think it would fit into my practice completely like that. (FCP3-16)

Some negative comments could not be addressed, such as concerns about the anti-CCP prediction model positive predictive value seeming low. Some comments were not addressed because they contradicted other participants’ feedback. For example, one participant felt the flowchart looked *a bit complicated* (GP3-18) and preferred the text version. In contrast, numerous participants reported finding the flowchart clear and helpful.

### Logic model

**Figure 3:**
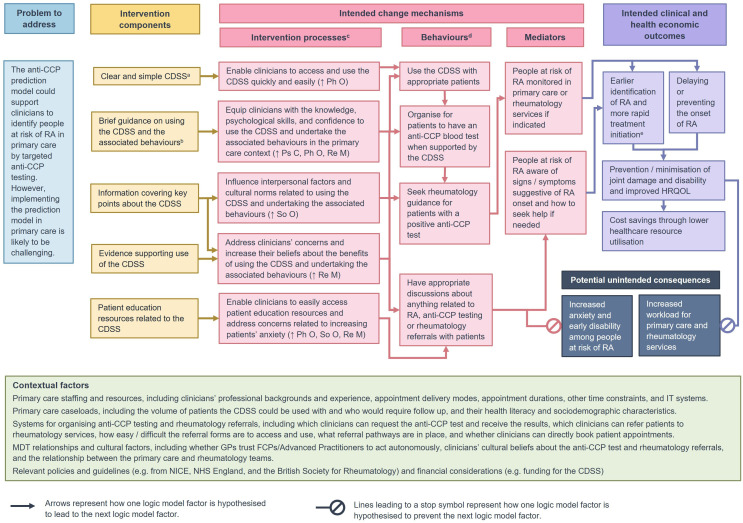
Could it Be RA? (COBRA) tool logic model V.3.0. Logic model summarising how the COBRA tool is intended to improve clinical and economic outcomes. Online supplemental appendix S7 provides an image description. ^a^The CDSS is a score chart based on the anti-CCP prediction model, which predicts whether a patient is likely to test positive for anti-CCP. ^b^The associated behaviours include all the clinician behaviours listed in the ‘Behaviours’ column. ^c^The text in brackets refers to the target component from the Capability Opportunity Motivation model of behaviour (COM-B).^43^ ^d^Refers to intended behaviours of primary care clinicians. ^e^Earlier identification of RA could include identification at the onset of overt signs and symptoms, and at the onset of subclinical inflammation identified through imaging. Anti-CCP, anti-cyclic citrullinated peptide; CDSS, clinical decision support system; FCP, first contact practitioner; GP, general practitioner; HRQOL, health-related quality of life; IT, information technology; MDT, multidisciplinary team; NHS, National Health System; NICE, National Institute for Health and Care Excellence; Ph O, physical opportunity; Ps C, psychological capability; RA, rheumatoid arthritis; Re M, reflective motivation; So O, social opportunity.

The programme theory of the COBRA tool after completion of the three development phases is presented as a logic model (V.3) in figure 3. This model summarises the intervention components and the processes by which they are intended to bring about a change to implement the prediction model in primary care settings. Contextual factors that might impact on future implementation and are important when considering future evaluation of the COBRA tool are also summarised.

## Discussion

Clinical prediction models to facilitate medical diagnosis and treatment decisions enable a personalised medicine approach in both rheumatology and primary care settings. Critical to their implementation in routine practice is to ensure that clinicians perceive the models are sufficiently robust to aid clinical decision-making, readily accessible and easily integrated into care. Our research included the views of clinicians (including GPs, FCPs and rheumatologists) and PPI representatives and was underpinned by a complex intervention research framework. In line with recommendations for CDSS development,^16^ it was critical to understanding the design of, and barriers and enablers to implementing the prediction model to identify people at risk of developing RA in primary care. To our knowledge, this level of detailed investigation to design and implement a CDSS, based on a rheumatology prediction model, is the first of its kind.

Drawing on behavioural change theory, with extensive input from key stakeholders, this three-phase study developed the COBRA tool in the form of a website. This tool incorporates a clinical prediction model into a format to support clinical decision-making. The COBRA tool is designed for use by primary care clinicians assessing patients presenting with new-onset MSK symptoms without synovitis to identify who might be anti-CCP positive and at risk of RA. Findings from each phase identified rich data informing when and how clinicians might use the prediction model and allowed us to optimise the content and user experience.

A strength of this study is that through understanding views of stakeholders, we developed and optimised the theory-informed COBRA tool. While the development process was time and resource intensive, with each phase, it identified key design issues that might influence primary care implementation and user-centred solutions. We employed purposive sampling and a range of recruitment approaches. This ensured participation of primary care clinicians operating in different regions, where the availability of anti-CCP testing and local rheumatology referral procedures varied. However, the majority of participants in phases 2 and 3 had requested an anti-CCP test in the last 12 months, which is contrary to survey data, indicating that less than half of GPs who would request tests to inform referral decisions for suspected RA would use anti-CCP antibody testing.^10^ Although our recruitment process allowed us to develop a CDSS that is likely to be applicable across regions and services, it remains possible that the self-selection of participants may introduce bias in favour of clinicians who might have more favourable views towards developing services to enhance rapid testing to identify patients at risk of developing RA.

While participants endorsed the acceptability and usability of the COBRA tool, we identified issues to future implementation, which might affect its adoption.

A key consideration for clinicians is when and how to use the tool within the time-pressured environment of primary care consultations. The COBRA tool has been developed for use with all patients with new-onset MSK symptoms, but our findings suggest this may not be appropriate or feasible in practice. Formal testing will determine if the COBRA tool is feasible for use in the intended population. Furthermore, although careful design of the website could reduce the likelihood that the COBRA tool is used in the wrong population (ie, patients with synovitis), there were still instances where clinicians were unsure if the tool should be used. This suggests that a short, one-off training package might be beneficial for new users to reinforce when and how to apply the COBRA tool and with whom.

The mode of delivery is important, and a key design requirement was that the CDSS must be in a readily accessible format necessitating a digital format. Some clinicians requested the CDSS be embedded within existing primary care electronic health systems, while others felt a web version was acceptable. Embedding the CDSS in different primary care services would require bespoke work with the different digital system providers. In this preliminary stage of development, we focused on using behavioural theory and user input to develop the content of the COBRA tool, which necessitated a web-based delivery within the time and resources available. We were also unable to test it within a routine service setting, which might identify different issues for clinicians and patients. Although this may prove a barrier to future implementation for some clinicians, future work is planned to explore how the website content might best be integrated into different electronic patient management systems, and to test the usability and acceptability of the COBRA tool in primary care consultations from both the clinician and patient perspective. The COBRA tool was not designed for direct patient use but as a clinical decision tool to support anti-CCP testing. The COBRA tool does, however, include resources intended for patients who are anti-CCP positive (table 6) that the clinician could provide (or signpost) to the patient for reference. Future work might include a self-report version for patients reporting new-onset MSK symptoms that could be completed in patient accessible health records, that is, the NHS app or Patients Know Best, prior to consultation with a primary care clinician.

Although some participants endorsed the content of the COBRA tool, primary care clinicians voiced concerns about whether or not rheumatology services would accept referrals for patients who are anti-CCP positive but without synovitis (ie, do not meet current referral guidance for RA).^37^ Survey data suggest anti-CCP-positive patients are being referred from primary care and seen by rheumatologists^38^; however, there is no guidance on how specialist services should manage patients at risk of developing RA. Participants felt that the success of implementing the COBRA tool was, in part, dependent on establishing a pathway of care across primary and specialist services. Although our PAG included input from rheumatology specialists, further work is needed to develop the management processes and referral pathways for individuals who are anti-CCP positive, which is acceptable to clinicians and patients.

## Conclusion

We conducted a three-phased process to develop a web-based tool to support clinician decision-making regarding targeted anti-CCP testing to identify individuals at risk of developing RA. Focusing on mitigating barriers to implementation, this in-depth design and evaluation allowed us to identify essential content and optimise the usability of the COBRA tool.

## Supplementary material

10.1136/rmdopen-2025-005957 online supplemental file 1

## Data Availability

No data are available.
