# Supplementary material for: Development of the ‘COuld it Be RA’ (COBRA) tool to facilitate early identification of people at risk of developing rheumatoid arthritis in primary care
Source: RMD Open. 2026 Feb 15;12(1):e005957. doi: 10.1136/rmdopen-2025-005957 (PMC12911672; doi:10.1136/rmdopen-2025-005957)
Supplement: online supplemental file 1 [file rmdopen-12-1-s001.docx]

Development of the ‘COuld it Be RA’ (COBRA) tool supplementary materials

# Supplementary Table S1: Key prediction model properties

| **Score threshold** | **% tested** | **PPV** | **NPV** | **Sens** | **Spec** | **No test** | | **Test** | | **% diagnosed with RA^a^** | |
| --- | --- | --- | --- | --- | --- | --- | --- | --- | --- | --- | --- |
|  |  |  |  |  |  | **CCP -ve**  **(true negatives)** | **CCP +ve**  **(false negatives)** | **CCP -ve**  **(false positives)** | **CCP +ve**  **(true positives)** | **No test** | **Test** |
| ≥ 8 | 40.38 | 4.39 | 98.02 | 60.10 | 60.22 | 4020 | 81 | 2656 | 122 | 0.27% | 1.33% |
| ≥ 11 | 18.72 | 6.06 | 97.76 | 38.42 | 81.88 | 5466 | 125 | 1210 | 78 | 0.32% | 2.33% |

^a^ Percentage of people diagnosed with RA by the 1-year follow up (follow-up data only available for 2480 (36%) participants and not rigorously quality checked). This data was added after completion of the first two interviews.

CCP, cyclic citrullinated peptide; NPV, negative predictive value; PPV, positive predictive value; RA, rheumatoid arthritis; sens, sensitivity; spec, specificity.

Table reproduced without modifications from Supplementary Table S1 in Anderson et al. (1) under the terms of the CC BY license (<https://creativecommons.org/licenses/by/4.0/>).

# Supplementary Table S2: Planned sample sizes

| **Phase** | **Sample size range** | **Aim guiding the final sample size** | **Rationale** |
| --- | --- | --- | --- |
| Phase 1: semi-structured interviews | 10–20 | Obtain sufficient data to provide rich insights into how primary care clinicians currently identify and refer patients with suspected RA, and the behaviours required to implement the anti-CCP prediction model in primary care. | Guidance for conducting a qualitative interview-based study within a larger project (2). |
| Phase 2: engagement workshops | 8–14 | Ensure the stakeholder group(s) are sufficiently diverse and feasible to manage. | The research team’s experience of group facilitation and previous relevant studies (3, 4). |
| Phase 3: think-aloud interviews | 6–12 | Reach a point at which ongoing qualitative data collection is unlikely to lead to the identification of additional substantial changes needed to refine the intervention. | Usability testing experiments (5) and previous relevant studies (6, 7). |

Anti-CCP, anti-cyclic citrullinated peptide; RA, rheumatoid arthritis.

# Supplementary Appendix S1: Intervention priorities

The Project Advisory Group (PAG) identified and discussed possible priorities for intervention during the first full PAG meeting. Feedback about the priorities was also received from a PAG member who was unable to attend the first meeting.

The priorities agreed by the PAG are listed below.

## Priorities

- Any digital components should be integrated within primary care IT systems (ideally fully integrated rather than a hyperlink to an external site).
- Multiple points for prompting clinicians about anti-cyclic citrullinated (anti-CCP) testing should be considered, including when clinicians submit rheumatoid factor test requests, enter read codes, and make referrals (e.g. via the DART form).
- Any components such as checklists must be simple and quick to use (e.g. three items rather than twelve).
- Any potentially ambiguous items in a checklist should be accompanied by explanations.
- There should be a strategy for making clinicians aware of the intervention and how to use it (e.g. by holding training sessions for GPs/trainees).
- The intervention should support clinicians to be transparent with patients (e.g. by sharing information visually), so that they can reach mutual agreement about testing and referral decisions.
- The intervention should encourage clinicians to provide patients with appropriate information (e.g. about how to arrange blood tests and what will happen after they have had them).

# Supplementary Appendix S2: Phase 2 topic guide

This Topic Guide is based on the ethically approved Phase 2 Indicative Topic Guide (version 1.0; 04 May 2023). The Topic Guide was finalised by the Project Management Group based on the Phase 1 findings and discussions with the Project Advisory Group.

## Workshop introduction

The facilitators should complete all the following actions at the start of the workshop.

1. Welcome everyone to the workshop and provide brief introductions.
2. Review key points form the Participant Information Sheet, including the following.

- The purpose of the workshop is to refine and prioritise a list of candidate components for a new intervention, the ‘Improving iDEntification of rheumatoid ArthritiS in primary care’ (IDEAS-PC) model.
- The aim of the IDEAS-PC model is to support clinicians to identify people at risk of rheumatoid arthritis (RA) in primary care through targeted anti-cyclic citrullinated peptide (anti-CCP) testing.
- The participants can withdraw at any time.
- The workshop will be recorded using Microsoft Teams and an encrypted digital voice recording device.
- The research team will keep each participant’s identifiable personal data confidential unless they identify any legal, safeguarding and/or safety concerns related to the participant, their patients and/or the researchers that require confidentiality to be breached.
- Fully anonymity cannot be guaranteed because other participants are present.
- The participants are required to keep the identities of all the other workshop participants, and the workshop discussions, confidential.
- The participants are required to respect each other’s views.

1. Remind any Project Advisory Group attendees (if present) that their role is to help with procedural trouble shooting and they cannot participate in the polls.
2. Offer the participants an opportunity to ask questions.
3. Confirm that the participants are happy to proceed.
4. Provide a brief presentation covering the workshop agenda, project background, and candidate components for the IDEAS-PC model.
5. Start recording the workshop using Microsoft Teams and an encrypted digital voice recording device.

## Workshop activities

The facilitators will lead the activities described below to help refine and prioritise the list of candidate components for the IDEAS-PC model. Depending on the progress of the workshop, some activities may be adapted or not completed. The potential prompts are for illustrative purposes and may not all be used.

### Activity 1: Additional components

Participants will be asked to identify any additional components they feel should be included in the IDEAS-PC model.

### Activity 2: Prioritisation Round 1

Participants will be asked to prioritise all the pre-specified candidate intervention components and any additional components suggested during Activity 1 using the Must have, Should have, Could have, Would like (MoSCoW) model^[[1]](#footnote-1)^. This will be achieved using one multiple choice Microsoft Teams poll per component (Appendix 1).

Prior to completing the prioritisation, participants will be shown a slide with the poll response options and the four points to consider for including each component in the IDEAS-PC model detailed in Activity 3a below. For each of the pre-specified candidate intervention components, participants will also be shown a summary slide of the component immediately before being asked to prioritise it.

### Activity 3: Candidate components discussion

Participants will be asked to discuss the candidate intervention components as described in Activities 3a and 3b below. The components will be prioritised based on the Activity 2 ratings as detailed in the analysis plan. The priority levels will be used to guide the discussions as shown in the table below.

| **Priority level** | **Discussion** |
| --- | --- |
| Must have | Discuss, focusing mainly on considerations for designing the component |
| Should have | Discuss, focusing mainly on considerations for including the component in the IDEAS-PC model |
| Could have | Do not discuss |
| Would like | Do not discuss |

If the workshop is split into smaller groups, the groups may discuss different components, with the aim of ensuring that all ‘Must have’ and ‘Should have’ components are discussed.

#### Activity 3a: Considerations for including the component in the IDEAS-PC model

Participants will be asked to discuss their views about whether to include the candidate intervention component in the IDEAS-PC model. To assist this, participants will be shown the following four points to consider for each component. These points are based on the APEASE criteria (affordability, practicability, effectiveness and cost-effectiveness, acceptability, side-effects/safety, and equity) detailed in the Behaviour Change Wheel approach^[[2]](#footnote-2)^.

1. How **affordable** is it likely to be?
2. How **practical** is it likely to be to deliver in routine practice in primary care?
3. How **acceptable** is it likely to be to clinicians?
4. What, if any, **concerns** do you have about its potential effectiveness/cost-effectiveness, safety, or impact on inequities?

#### Activity 3b: Considerations for designing the component

Participants will be asked to discuss considerations for designing the candidate intervention component. To assist this, participants will be shown the following two points to consider for each component.

1. **What** do you think it should include?
2. How do you think it should be **delivered?**

### Activity 4: Combining, implementing and embedding discussion

Participants will be asked to discuss how the intervention components should be combined, implemented, and embedded into routine practice in primary care. To assist this, participants will be shown the following three points to consider.

1. How do you think the components should be **combined?**
2. How do you think the components should be **put into practice** in primary care?
3. What do you think would help make using the components become a **normal part of routine practice** in primary care?

### Activity 5: Prioritisation Round 2

Participants will be asked to prioritise all the candidate intervention components using the same approach as in Activity 2.

## Workshop closure

The facilitators should complete all the following actions at the end of the workshop.

1. Thank the participants for taking part in the workshop.
2. Offer the participants an opportunity to ask questions.
3. Confirm the plans for providing the £60 recognition payment and Certificate of Participation.

## Appendix 1: Prioritisation poll

A separate multiple-choice poll will be used for each candidate intervention component. The polls will be labelled with the names of the candidate intervention components detailed below. Each poll will include four response options: ‘Must have’, ‘Should have’, ‘Could have’ and ‘Would like’.

1. Decision tool
2. Evidence supporting use of the decision tool
3. Guidance on using the decision tool and associated actions
4. Information covering key points about the decision tool
5. Video of and/or quotes from MDT professionals supporting use of the decision tool and associated actions
6. Reminders about using the decision tool
7. Summary information about the decision tool for sharing via appropriate routes
8. Clinical audit tool for the decision tool
9. Patient education resources

The Round 1 polls will be labelled ‘1.1 Decision tool’, ‘1.2 Evidence supporting use of the decision tool’ etc. The Round 2 polls will be labelled ‘2.1 Decision tool’, ‘2.2 Evidence supporting use of the decision tool’ etc.

Extra polls using the same format will be added for any additional components suggested in Activity 1.

# Supplementary Appendix S3: Phase 3 topic guide

This Topic Guide will be used for all the Phase 3 interviews. During the interviews the IDEAS-PC model prototype will be referred to by its public-facing name, the ‘COuld it Be RA?’ (COBRA) tool, which was suggested and agreed by the Project Advisory Group.

Each interview will include two parts. During Part 1, the participant will be asked to work through the IDEAS-PC model prototype whilst saying everything they are thinking out loud. The interviewer may intervene when appropriate e.g., to guide the participant to specific content or ask them probing questions about the prototype’s content and usability. Fictional clinical vignettes may be used to support participants to work through the IDEAS-PC model if appropriate. Example fictional clinical vignettes are available in a separate document. During Part 2, the interviewer will ask the participant brief semi-structured interview questions to explore their overall perspectives of the IDEAS-PC model prototype.

Potential prompts for both parts of the interview are provided below. The prompts are for illustrative purposes only and may not all be used. Additional prompts may be used where appropriate.

The Topic Guide will be pilot tested with at least one clinician member of the Project Advisory Group once the IDEAS-PC model prototype is available. Minor wording amendments may be made to the Topic Guide based on the pilot testing process if required. If any substantial amendments are required, the amended Topic Guide will be submitted for ethical review as an amendment.

The Topic Guide may be amended during the data collection phase so that issues identified in earlier interviews can be explored in subsequent interviews if appropriate. Amendments will only be made if SR, HS and AA agree that they are important.

## Interview introduction

The interviewer should complete all the following actions at the start of each interview.

1. Review key points form the Participant Information Sheet, including the following.

- The purpose of the interview is to explore how easy the ‘COuld it Be RA?’ (COBRA) tool is to use and what clinicians think of it, so that the tool can be gradually improved.
- The COBRA tool is still in development, so participants will not be asked to use it with real patients at any point in the study.
- Critical feedback about the COBRA tool will be particularly useful for improving it.
- The purpose of the interview is not to test the participant’s knowledge and there are no right or wrong answers.
- The participant can withdraw at any time.
- The interview will be recorded using two approaches.
- The participant’s identifiable personal data will be kept confidential unless the research team identify any legal, safeguarding and/or safety concerns related to the participant, their patients and/or the researchers that require confidentiality to be breached.

1. Explain the process for the interview, including the following.

- The interview will include two parts.
- During Part 1, the participant should say everything they are thinking out loud as they work through the COBRA tool.
- As part of that process, the participant may find it helpful to read text out loud and say what they are clicking on and why.
- The interviewer may ask the participant prompt questions and direct them to specific parts of the COBRA tool.
- The interviewer may ask the participant to try using the COBRA tool with fictional patient cases.
- During Part 2, the interviewer will ask participant about their overall views of the COBRA tool.
- If the participant asks the interviewer questions about the COBRA tool, the interviewer may not be able to discuss the questions until the end of the interview.

1. Offer the participant an opportunity to ask questions.
2. Confirm that the participant is happy to proceed.
3. Start the recording using two approaches.

## Part 1: Think-aloud interview prompts

[The prompts are for illustrative purposes only and may not all be used. Additional prompts may be used where appropriate.]

- What do you think about the <<COBRA tool feature>>?
- How would you feel about using <<COBRA tool feature>> in your clinical practice?
- Can you tell me about anything you particularly like about <<COBRA tool feature>>?
- Can you tell me about anything you particularly do not like about <<COBRA tool feature>>?
- What are you thinking now?
- Why did you select <<COBRA tool feature>>?
- Can you tell me about whether you would use the COBRA tool for this patient [described in the fictional clinical vignette]?
- Can you explain what you do next if you were using the COBRA tool in your clinical practice and why?

## Part 2: Semi-structured interview prompts

[The prompts are for illustrative purposes only and may not all be used. Additional prompts may be used where appropriate.]

- What are your overall views of the COBRA tool?
- Can you tell me about whether you think you would use the COBRA tool in your clinical practice?
- How do you think using the COBRA tool would affect your interactions with patients?
- Can you tell me about any parts of the COBRA tool you think we should change?
- Is there anything else you would like to add?

## Interview closure

The interviewer should complete all the following actions at the end of each interview.

1. Thank the participant for taking part in the interview.
2. Offer the participant an opportunity to ask questions.
3. Confirm the plans for providing the £60 recognition payment (and travel expenses if applicable) and Certificate of Participation.

# Supplementary Table S3: Example fictional clinical vignettes

Five vignettes were developed at the start of the study. Based on the Phase 3 interview findings, a further four vignettes were added, two of which were subsequently refined. This enabled issues identified in earlier interviews to be explored in later interviews.

| **Patient case 1** | |
| --- | --- |
| History | 57-year-old male with right shoulder pain, which started three months ago after a particularly busy time with his building job.  The pain has been coming and going but seems to be getting worse overall. |
| Family history of RA | Mother has RA. |
| Smoking status | Smoked between the ages of 17 and 30 years-old.  Does not smoke now. |
| Key objective findings | Objective assessment not possible (telephone consultation). |
| **Patient case 6c** | |
| History | 37-year-old female with pain in both wrists and hands and across the balls of her feet. The pain started gradually two to three months ago and is getting gradually worse.  Cannot think of any cause for her symptoms. |
| Family history of RA | None. |
| Smoking status | Vapes but has never smoked cigarettes. |
| Key objective findings | Swelling in both wrists and across the balls of her feet. Positive squeeze test for the MCP joints. Feet not examined. |
| **Patient case 7b** | |
| History | 18-year-old male with right shoulder and elbow pain.  Pain started gradually a couple of months ago. Cannot remember injuring his arm. |
| Family history of RA | Unknown (care leaver) |
| Smoking status | Smoked between the ages of 14 and 16-years old.  Does not smoke now. |
| Key objective findings | Objective assessment not possible (telephone consultation). |
| **Patient case 8** | |
| History | 51-year-old female who has been experiencing achy shoulders and hips and painful feet for around two months. Has also been experiencing hot flushes for a few months. |
| Family history of RA | Brother has RA. |
| Smoking status | Gave up smoking when she had her daughter 15 years ago. |
| Key objective findings | No evidence of swelling or stiffness. Joint symptoms not aggravated by movement, strength testing or palpation. |

MCP, metacarpophalangeal; RA, rheumatoid arthritis.

# Supplementary Table S4: Behavioural analysis for using the CDSS with appropriate patients

| **Target component(s) (COM-B)** | **Set of barriers [B] and facilitators [F] to the target behaviour** | **Candidate components** | **Intervention function(s) (BCW)** | **Behaviour change technique(s) (BCTTv1)** | **Themes^1^** |
| --- | --- | --- | --- | --- | --- |
| Psychological capability | Forgetting to use the CDSS [B]  Lack of awareness of the CDSS [B] | Electronic CDSS integrated into EHR systems and templates.  Email reminders to use the CDSS.  EHR pop-up reminders to use the CDSS.  Reminders to use the CDSS on relevant forms e.g. for requesting blood tests, entering read codes and making referrals.  Reminders about the CDSS for sharing during clinician meetings and training sessions.  Reminders on posters and mouse mats for clinical areas. | Environmental restructuring | 7.1 Prompts/cues  8.3 Habit formation  12.5 Adding objects to the environment | Access to and usability of the CDSS |
|  | Not fully understanding how to assess the predictor variables [B]  Uncertainty about which patients to use the CDSS with [B]  Understanding which patients to use the CDSS with [F] | Guidance on how to use the CDSS, including how to assess the predictor variables (e.g. whether to assess current pain or pain over a specific timescale) and which patients to use / not use the CDSS, provided through formats such as:   - brief text - 5 to 10-minute video on how to use the CDSS (e.g. of a clinician demonstrating how to use the CDSS) - case studies demonstrating when it is appropriate to use / not use the CDSS (which could be used for team discussions) | Training  Modelling | 4.1 Instruction on how to perform the behaviour  6.1 Demonstration of behaviour  9.1 Credible source |  |
| Physical opportunity | Large volume of patients who the CDSS could be used with [B] | N/A (contextual moderator) | N/A | N/A | Variations in current practice |
|  | Patients having multiple concerns to discuss [B] | N/A (contextual moderator) | N/A | N/A |  |
|  | Accessing the CDSS being time consuming or difficult [B]  Accessing the CDSS being quick and easy [F] | CDSS available in more than one format, including:   - electronic format integrated with EHR systems and templates, which ideally autosaves into patients’ notes and is potentially available as a pop-up - standalone website, which is ideally easily accessible via a link or standard search engine - printable / laminated sheet | Environmental restructuring | 12.1 Restructuring the physical environment  12.5 Adding objects to the environment | Access to and usability of the CDSS |
|  | Using the CDSS being time consuming or difficult [B]  Using the CDSS being quick and easy [F] | Brief guidance on how to use the CDSS  Clear and simple CDSS that:   - includes one or two tables with the predictor variables and tick boxes, potentially with an optional manikin - lists the predictor variables in a logical order (e.g. FDR first, then sex, smoking history and the joint pain variables from head to toe) **OR** in an order that means it may not be necessary to score them all, along with an explanation about why they are listed that way - is available in an electronic format with automatic and transparent scoring (potentially with the individual item scores removed or just in brackets) - can be completed directly by clinicians (ideally with an optional patient questionnaire for some circumstances) - provides recommended actions for the scores obtained | Training  Environmental restructuring | 4.1 Instruction on how to perform the behaviour  12.1 Restructuring the physical environment |  |
| Social opportunity | Concerns about GPs perceiving FCPs are requesting too many anti-CCP tests [B] | Information highlighting who the intended users of the CDSS are.  Information highlighting GPs were involved in developing the CDSS.  Video of and/or quotes from GPs encouraging other clinicians to use the CDSS. | Education  Persuasion | 5.3 Information about social and environmental consequences  6.3 Information about others’ approval  9.1 Credible source | Interpersonal influences across the whole healthcare system |
|  | Opportunity to use the CDSS to support patient education [F] | Information highlighting that clinicians can show the CDSS to patients to support patient education and involve patients in their care.  Electronic CDSS with automatic and transparent scoring. | Education  Environmental restructuring | 5.3 Information about social and environmental consequences  12.1 Restructuring the physical environment |  |
| Reflective motivation | Concerns that using the CDSS may have negative consequences for patients [B] | Information highlighting that providing appropriate education can help address patient anxiety.  Guidance on how to discuss the CDSS and related points (e.g. implications of receiving a positive anti-CCP test result) with patients.  Patient education resources, such as a leaflet that includes information about:   - the purpose of the anti-CCP test - what the test results mean, including potential implications such as affecting insurance policies - how the test results will be used/followed up - why patients may be referred rheumatology services (including the potential benefits of referral) - lifestyle advice - safety netting advice (including for if patients have ongoing or worsening symptoms) | Education  Training  Environmental restructuring | 4.1 Instruction on how to perform the behaviour  5.1 Information about health consequences  5.3 Information about social and environmental consequences  5.6 Information about emotional consequences  12.5 Adding objects to the environment | Potential risks and benefits |
|  | Concerns about managing people at risk of RA [B] | Information and evidence about potential referral, monitoring and intervention options for people at risk of RA. | Education | 5.1 Information about health consequences  5.6 Information about emotional consequences  5.3 Information about social and environmental consequences |  |
|  | Lack of concerns about the workload implications of using the CDSS [F]  Concerns about the workload implications for the whole system [B] | Video of and/quotes from multidisciplinary professionals (e.g. rheumatologists, GPs, FCPs, nurses and phlebotomists) reinforcing that using the CDSS is appropriate from a workload perspective.  CDSS based on the 11-point threshold (so that fewer patients are tested than if the CDSS was based on the 8-point threshold). | Persuasion  Environment restructuring | 5.3 Information about social and environmental consequences  6.3 Information about others’ approval  9.1 Credible source  12.1 Restructuring the physical environment |  |
|  | Concerns about missing patients who should be referred to rheumatology services [B] | Information highlighting:   - people who are anti-CCP negative may still develop RA - the CDSS is just a decision aid and blood tests are just one part of the clinical picture   Patient education resources such as a leaflet that include safety netting advice.  CDSS based on the 8-point threshold (so that there are more true positives / fewer false negatives than if it was based on the 11-point threshold). | Education  Environment restructuring | 5.1 Information about health consequences  12.1 Restructuring the physical environment  12.5 Adding objects to the environment |  |
|  | Uncertainty about whether using the CDSS will change practice [B]  Believing certain patients (e.g. those without classical RA symptoms) are unlikely to test positive for anti-CCP [B]  Concerns about cost implications of using the CDSS [B]  Concerns about the predictor variables, prediction model and/or supporting evidence base [B]  Perceiving the anti-CCP test has limited value [B]  Believing the predictor variables and/or prediction model look appropriate [F]  Believing there are benefits of the anti-CCP test [F]  Believing the CDSS will support clinical reasoning [F]  Believing using the CDSS will have benefits for patients [F]  Believing early identification of RA will improve patient outcomes [F] | Information highlighting the potential benefits of using the CDSS, including for supporting clinical reasoning and improving patient outcomes.  Brief summary of the purpose of the CDSS and its potential benefits that can be shared through appropriate routes (e.g. TARGET sessions, NICE guidelines, or courses like Red Whale).  Clinical audit tool that supports clinicians to record their use of the CDSS and if / how it affected their practice.  Evidence supporting use of the CDSS, including evidence about:   - the diagnostic test properties of the anti-CCP test - how the anti-CCP prediction model was developed - the anti-CCP prediction model properties (ideally with data on the proportion of patients who develop RA with a year) - results of the health economic modelling (ideally incorporating whether patients would have had the anti-CCP blood test if the CDSS had not been used and whether they had any other blood tests) - potential benefits and risks of identifying people at risk of RA and potential referral, monitoring and intervention options | Education  Persuasion  Incentivisation | 2.3 Self-monitoring of behaviour  2.4 Self-monitoring of outcome(s) of behaviour  5.1 Information about health consequences  5.3 Information about social and environmental consequences  5.6 Information about emotional consequences  9.1 Credible source |  |
| Automatic motivation | Getting into a habit of using the CDSS [F]  Having prompts to use the CDSS [F] | Electronic CDSS integrated into EHR systems and templates.  Email reminders to use the CDSS.  EHR pop-up reminders to use the CDSS.  Reminders to use the CDSS on relevant forms e.g. for requesting blood tests, entering read codes and making referrals.  Reminders about the CDSS for sharing during clinician meetings and training sessions.  Reminders on posters and mouse mats for clinical areas. | Environmental restructuring | 7.1 Prompts/cues  8.3 Habit formation  12.5 Adding objects to the environment | Access to and usability of the CDSS |

Anti-CCP, anti-cyclic citrullinated peptide; BCW, Behaviour Change Wheel (8, 9); BCTTv1, Behaviour Change Technique Taxonomy (v1) (10); CDSS, clinical decision support system; COM-B, Capability Opportunity Motivation model of behaviour (8); EHR, electronic health record; FCP, First Contact Practitioner; FDR, first degree relative; RA, rheumatoid arthritis.

^1^Themes from Anderson et al. (1).

# Supplementary Table S5: Behavioural analysis for organising for patients to have an anti-CCP test when supported by the CDSS

| **Target component(s) (COM-B)** | **Set of barriers [B] and facilitators [F] to the target behaviour** | **Candidate components** | **Intervention function(s) (BCW)** | **Behaviour change technique(s) (BCTTv1)** | **Themes^1^** |
| --- | --- | --- | --- | --- | --- |
| Psychological capability | Lack of awareness of the anti-CCP test on the blood test requesting system [B] | Guidance on how to organise an anti-CCP blood test, including that it is likely to be available on the blood test requesting system. | Training | 4.1 Instruction on how to perform the behaviour | Variations in current practice |
| Physical opportunity | Guidance intended to limit use of the anti-CCP test [B] | Video of and/or quotes from multidisciplinary professionals (e.g. rheumatologists, GPs, and FCPs) reinforcing that it is appropriate to organise for patients to have anti-CCP test when supported by the CDSS. | Persuasion | 5.3 Information about social and environmental consequences  6.3 Information about others’ approval  9.1 Credible source |  |
|  | Not being able to directly request anti-CCP blood tests  Being able to directly request anti-CCP tests [F] | N/A (contextual moderator) | N/A | N/A |  |
| Social opportunity | Choice of blood tests being left to the direct requester [B]  Choice of blood tests being specified by the assessor [F] | Guidance on how to organise an anti-CCP blood test, including how to effectively task a direct requestor about the anti-CCP test by:   - explicitly stating the anti-CCP test is required - justifying why the anti-CCP test is required. | Training | 4.1 Instruction on how to perform the behaviour | Interpersonal influences across the whole healthcare system |
|  | GPs not trusting FCPs to act autonomously [B]  GPs trusting FCPs to act autonomously [F] | N/A (contextual moderator) | N/A | N/A |  |

Anti-CCP, anti-cyclic citrullinated peptide; BCW, Behaviour Change Wheel (8, 9); BCTTv1, Behaviour Change Technique Taxonomy (v1) (10); CDSS, clinical decision support system; COM-B, Capability Opportunity Motivation model of behaviour (8); FCP, First Contact Practitioner.

^1^Themes from Anderson et al. (1).

# Supplementary Table S6: Behavioural analysis for referring patients with a positive anti-CCP test to rheumatology services

| **Target component(s) (COM-B)** | **Set of barriers [B] and facilitators [F] to the target behaviour^1^** | **Candidate components** | **Intervention function(s) (BCW)** | **Behaviour change technique(s) (BCTTv1)** | **Themes^1^** |
| --- | --- | --- | --- | --- | --- |
| Psychological capability | Limited knowledge about which patients are appropriate to refer to rheumatology [B] | Guidance on which patients are appropriate to refer to rheumatology services and why (e.g. based on an anti-CCP threshold value and any other relevant signs/symptoms/test results). | Education  Training | 4.1 Instruction on how to perform the behaviour  5.1 Information about health consequences | Variations in current practice |
|  | Being unsure about some rheumatology referral procedures [B] | Guidance on potential approaches for making a rheumatology referral. | Training | 4.1 Instruction on how to perform the behaviour |  |
| Physical opportunity | Not directly receiving anti-CCP test results [B] | N/A (contextual moderator). | N/A | N/A |  |
|  | Referral process being time-consuming or difficult [B]  Referral process being quick and easy [F] | N/A (contextual moderator). | N/A | N/A |  |
|  | Not being able to directly refer patients to rheumatology services [B]  Not being able to directly book patients’ appointments [B]  Being able to directly refer patients to rheumatology services [F]  Being able to directly book patients’ appointments [F] | N/A (contextual moderator). | N/A | N/A |  |
| Social opportunity | Potential lack of acceptance of referrals by the rheumatology team [B] | Video of and/or quotes from rheumatologists reinforcing that they will accept patients with a positive anti-CCP test even if they do not have classical symptoms of RA. | Persuasion | 5.3 Information about social and environmental consequences  6.3 Information about others’ approval  9.1 Credible source | Interpersonal influences across the whole healthcare system |
|  | Patient wants a rheumatology referral [F] | Patient education resources, such as a leaflet, that include information about the potential benefits of being referred to rheumatology services. | Education  Environment restructuring | 5.1 Information about health consequences  12.1 Restructuring the physical environment  12.5 Adding objects to the environment |  |
|  | Positive relationships, experiences, and feedback from the rheumatology team [F] | N/A (contextual moderator). | N/A | N/A |  |
|  | GPs and FCPs undertaking shared decision making about how to follow up on test results [F] | Information highlighting it may be helpful for GPs and FCPs to collaboratively decide how to follow-up on anti-CCP test results. | Education | 3.1 Social support (unspecified) |  |
| Reflective motivation | Concerns about inappropriately burdening rheumatology services and increasing waiting times [B]  Believing patients who are anti-CCP positive should be referred to rheumatology services [F] | Video of and/or quotes from rheumatologists reinforcing that referring patients with a positive anti-CCP test is appropriate and unlikely to overburden their services or increase waiting times. | Persuasion | 5.3 Information about social and environmental consequences  6.3 Information about others’ approval  9.1 Credible source | Potential risks and benefits |

Anti-CCP, anti-cyclic citrullinated peptide; BCW, Behaviour Change Wheel (8, 9); BCTTv1, Behaviour Change Technique Taxonomy (v1) COM-B, Capability Opportunity Motivation model of behaviour (8); FCP, First Contact Practitioner.

^1^Themes from Anderson et al. (1).

# Supplementary Table S7: Behavioural analysis for having appropriate discussions about anything related to RA, anti-CCP testing or rheumatology referrals with patients

| **Target component(s) (COM-B)** | **Set of barriers [B] and facilitators [F] to the target behaviour^1^** | **Candidate components** | **Intervention function(s) (BCW)** | **Behaviour change technique(s) (BCTTv1)** | **Themes^1^** |
| --- | --- | --- | --- | --- | --- |
| Psychological capability | Not knowing how long the rheumatology waiting times will be [B] | Guidance on how to obtain information about rheumatology waiting times for providing to patients. | Training | 4.1 Instruction on how to perform the behaviour | Interpersonal influences across the whole healthcare system |
|  | Having the knowledge and skills to appropriately communicate points about RA, anti-CCP testing or rheumatology referrals to patients [F]  Having the knowledge and skills to involve patients in decision-making [F] | Guidance on how clinicians can apply their existing knowledge and skills when communicating about / undertaking shared decision making related to the CDSS. | Training | 4.1 Instruction on how to perform the behaviour |  |
| Physical opportunity | Limited time to have discussions with patients [B]  Having easy access to patient information resources [F]  Having quick and easy options for contacting patients e.g., via text [F] | Guidance on how to briefly discuss key points about / related to the CDSS with patients (e.g. how to arrange blood tests and how the test results will be used/followed up).  Easily accessible patient education resources, such as a leaflet that can be shared via text. | Training  Environmental restructuring | 4.1 Instruction on how to perform the behaviour  11.3 Conserving mental resources  12.5 Adding objects to the environment | Interpersonal influences across the whole healthcare system |
|  | Low numbers of patients to follow up about anti-CCP testing [F] | N/A (contextual moderator) | N/A | N/A | Variations in current practice |
| Social opportunity | Assuming patients will want blood tests and a rheumatology referral [B]  Perceiving it is normal practice not to involve patients in decisions about whether to have blood tests [B] | Information highlighting the importance of involving patients when requesting blood tests and undertaking rheumatology referrals. | Education | 5.3 Information about social and environmental consequences | Interpersonal influences across the whole healthcare system |
|  | Perceiving there is no negative stigma associated with RA [F] | Information highlighting that there is no negative stigma associated with RA. | Education | 5.3 Information about social and environmental consequences |  |
| Reflective motivation | Perceiving there is limited choice about whether to refer some patients to rheumatology services [B] | Information highlighting the importance of involving patients when undertaking rheumatology referrals. | Education | 5.3 Information about social and environmental consequences | Potential risks and benefits |
|  | Believing too much information may have negative consequences for patients [B]  Believing discussions will be helpful and/or reassuring for patients [F] | Guidance on how to help ensure patient discussions are helpful and reassuring for patients, for example by explaining:   - only a small proportion of patients who have an anti-CCP test will test positive - only a small proportion of patients who test positive for anti-CCP will develop RA - patients can proactively optimise their health through lifestyle changes - early identification of RA improves patient outcomes - effective management options are available for RA, and the prognosis is now much better than it used to be. | Training | 4.1 Instruction on how to perform the behaviour  5.1 Information about health consequences  5.6 Information about emotional consequences |  |
|  | Believing some patients may want to find out if they are at risk of RA [F] | Information highlighting some people may want to find out if they are at risk of RA. | Education | 5.3 Information about social and environmental consequences |  |

Anti-CCP, anti-cyclic citrullinated peptide; BCW, Behaviour Change Wheel (8, 9); BCTTv1, Behaviour Change Technique Taxonomy (v1) (10); CDSS, clinical decision support system; COM-B, Capability Opportunity Motivation model of behaviour (8).

^1^Themes from Anderson et al. (1).

# Supplementary Table S8: Initial list of candidate components

| **Candidate components** | **Design considerations based on the Phase 1 findings and Project Advisory Group discussions** |
| --- | --- |
| 1. CDSS | Ensure the CDSS is clear and simple.  Base the CDSS on the 11-point threshold (so fewer patients are tested) **OR** the 8-point threshold (so there are more true positives / fewer false negatives) – agreed with the PAG that using the 11-point threshold would be more appropriate due to the cost and workload implications.  Provide the CDSS in more than one format, including:   - electronic format integrated with EHR systems and templates, which has automatic and transparent scoring (potentially with the individual item scores removed or just in brackets), ideally auto-populates some of the variables (e.g. sex), and autosaves into patients’ notes, and is potentially available as a pop-up - standalone website, which is ideally easily accessible via a link or standard search engine - printable/laminated sheet   Ensure the CDSS can be completed directly by clinicians (ideally with an optional patient questionnaire for some circumstances).  Include one or two tables with the predictor variables and tick boxes, potentially with an optional manikin.  List the predictor variables in a logical order (e.g. FDR first, then sex, smoking history and the joint pain variables from head to toe) **OR** in an order that means it may not be necessary to score them all, along with an explanation about why they are listed that way – agreed with the PAG that listing the variables in a logical order would be best with first degree relative at the top and then the joint pain variables listed from head to toe. |
| 1. Evidence supporting use of the CDSS | Provide evidence about:   - how the anti-CCP prediction model was developed - the anti-CCP prediction model properties (ideally with data on the proportion of patients who develop RA with a year) - results of the health economic modelling (ideally incorporating whether patients would have had the anti-CCP blood test if the CDSS had not been used and whether they had any other blood tests) - the diagnostic test properties of the anti-CCP test - potential benefits and risks of identifying and people at risk of RA and potential referral, monitoring and intervention options |
| 1. Guidance on using the CDSS and undertaking the associated behaviours | Provide brief guidance on using the CDSS and follow-up actions that covers:   - which patients to use / not use the CDSS with - how to assess the predictor variables (e.g. whether to assess current pain or pain over a specific timescale) - how to organise an anti-CCP test, including that it is likely to be available on the blood test requesting system and how to effectively task a direct requestor by:   - explicitly stating the anti-CCP test is required   - justifying why the anti-CCP test is required - which patients are appropriate to refer to rheumatology services and why (e.g. based on an anti-CCP threshold value and any other relevant signs/symptoms/test results) - potential approaches for making a rheumatology referral - if/when the anti-CCP test should be repeated   Provide guidance on patient education that covers:   - how to briefly discuss key points about / related to the CDSS with patients (e.g. how to arrange blood tests, how the test results will be used/followed up, implications of receiving a positive anti-CCP test result, safety-netting advice) - how clinicians can apply their existing knowledge and skills when communicating about / undertaking shared decision making related to the CDSS - how to obtain information about rheumatology waiting times for providing to patients - how to help ensure patient discussions are helpful and reassuring for patients, for example by explaining:   - only a small proportion of patients who have an anti-CCP test will test positive   - only a small proportion of patients who test positive for anti-CCP will develop RA   - patients can proactively optimise their health through lifestyle changes   - early identification of RA improves patient outcomes   - being referred to rheumatology services has benefits   - effective management options are available for RA, and the prognosis is now much better than it used to be   Provide guidance through formats such as:   - brief text - flow chart - 5 to 10-minute video (e.g. of a clinician demonstrating how to use the CDSS) - case studies demonstrating when it is appropriate to use / not use the CDSS (which could be used for team discussions) |
| 1. Information covering key points about the CDSS | Provide information that highlights:   - who the intended users of the CDSS are - potential benefits of using the CDSS, including for supporting clinical reasoning and improving patient outcomes - potential referral, monitoring and intervention options for people at risk of RA - GPs were involved in developing the CDSS - it may be helpful for GPs and FCPs to collaboratively decide how to follow-up on anti-CCP test results - people who are anti-CCP negative may still develop RA - the CDSS is just a decision aid and blood tests are just one part of the clinical picture – highlighted as particularly important by the PAG. - there is no negative stigma associated with RA - some people may want to find out if they are at risk of RA - clinicians can show the CDSS to patients to support patient education and involve patients in their care - providing appropriate education can help address patient anxiety - the importance of involving patients when requesting blood tests and undertaking rheumatology referrals |
| 1. Video of and/or quotes from multi-disciplinary professionals supporting use of the CDSS and associated behaviours | Include the following professionals and points:   - GPs encouraging other clinicians to use the CDSS - multidisciplinary professionals (e.g. rheumatologists, GPs, FCPs, nurses and phlebotomists) reinforcing that using the CDSS is appropriate from a workload perspective - multidisciplinary professionals (e.g. rheumatologists, GPs, and FCPs) reinforcing that it is appropriate to organise for patients to have anti-CCP test when supported by the CDSS - rheumatologists reinforcing that referring patients with a positive anti-CCP test is appropriate and unlikely to overburden their services or increase waiting times, and that they will accept patients with a positive anti-CCP test even if they do not have classical symptoms of RA |
| 1. Reminders about using the CDSS | Provide reminders in formats such as:   - email reminders - EHR pop-up reminders - reminders on relevant forms e.g. for requesting blood tests, entering read codes and making referrals - reminders about the CDSS for sharing during clinician meetings and training sessions - reminders on posters and mouse mats for clinical areas |
| 1. Summary information about the CDSS for sharing via appropriate routes | Include brief information about the purpose of the CDSS and its potential benefits.  Share the information via routes such as:   - TARGET sessions - NICE guidelines - Courses like Red Whale |
| 1. Clinical audit tool | Support clinicians to record their use of the CDSS and if / how it affected their practice. |
| 1. Patient education resources | Provide easily accessible patient education resources such as a leaflet that can be shared via text.  Include information about:   - the purpose of the anti-CCP test - what the test results mean, including potential implications such as affecting insurance policies - how the test results will be used / followed up - why patients may be referred rheumatology services (including the potential benefits of referral) - lifestyle advice - safety netting advice (including for if patients have ongoing or worsening symptoms) |

Anti-CCP, anti-cyclic citrullinated peptide; CDSS, clinical decision support system; EHR, electronic health record; FCP, First Contact Practitioner; FDR, first degree relative; GP, general practitioner; PAG, Project Advisory Group; RA, rheumatoid arthritis

# Supplementary Table S9: Phase 2 participant characteristics

| **Characteristic^1^** | **N (%)** |
| --- | --- |
| **Professional role** | |
| GP | 3 (38) |
| FCP with a physiotherapy background | 5 (63) |
| **Years’ experience in current role** | |
| < 5 | 3 (38) |
| 5 < 15 | 3 (38) |
| ≥ 15 | 2 (25) |
| **Type(s) of organisation^2^** | |
| General practice | 4 (50) |
| GP confederation | 1 (13) |
| NHS community trust | 3 (38) |
| Primary care network | 1 (13) |
| **Able to request the anti-CCP test^2^** | |
| Yes directly | 4 (50) |
| Yes but only if obtains approval from rheumatology | 1 (13) |
| Yes via another professional | 4 (50) |
| **Requested an anti-CCP blood test in the past 12 months** | |
| Yes | 7 (88) |
| No | 1 (13) |
| **Experience of recruiting participants to a previous primary care anti-CCP testing study (11)** | |
| Yes | 1 (13) |
| No | 7 (88) |
| **Integrated Care System area where workplace is located** | |
| Bath and North East Somerset, Swindon and Wiltshire | 1 (13) |
| Cornwall and Isles of Scilly | 1 (13) |
| Humber and North Yorkshire | 1 (13) |
| West Yorkshire | 5 (63) |
| **Recruitment approach** | |
| NHS community trust | 3 (38) |
| Advertisement shared via professional network | 4 (50) |
| Snowball recruitment | 1 (13) |

^1^ Characteristics are only reported for roles in which clinicians undertake regular weekly clinics for a provider of NHS primary care services, which include assessing people with new-onset MSK symptoms without synovitis.

^2^ Participants could report more than one option.

Anti-CCP, anti-cyclic citrullinated peptide; FCP, First Contact Practitioner; NHS, National Health Service.

# Supplementary Table S10: Priority ratings summary

| **Candidate component** | **Overall ratings (n=8)** | | **Workshop 1 (n=5)** | | **Workshop 2 (n=3)** | |
| --- | --- | --- | --- | --- | --- | --- |
|  | **Round 1** | **Round 2** | **Round 1** | **Round 2** | **Round 1** | **Round 2** |
| CDSS^a^ | Must have^b^ | Must have^b^ | Must have^b^ | Must have^b^ | Must have^b^ | Must have^b^ |
| Evidence supporting use of the CDSS^a^ | Should have | Should have^b^ | Should have | Should have^b^ | Should have | Should have |
| Guidance on using the CDSS and undertaking the associated behaviours^a^ | Must have^b^ | Must have^b^ | Must have^b^ | Should have | Should have | Must have^b^ |
| Information covering key points about the CDSS^a^ | Could have | Could have | Should have | Should have^b^ | Could have^b^ | Could have |
| Video of and/or quotes from MDT professionals | Could have^b^ | Could have^b^ | Could have^b^ | Could have^b^ | Would like | Could have^b^ |
| Reminders about using the CDSS | Should have | Could have | Should have^b^ | Could have | Would like | Would like |
| Summary information about the CDSS | Should have | Could have | Should have | Should have | Would like | Would like |
| Clinical audit tool | Could have | Could have | Could have | Could have | Would like | Could have |
| Patient education resources^a^ | Should have | Should have | Could have | Should have^a^ | Should have | Could have |

CDSS, clinical decision support system; MDT, multi-disciplinary.

^a^ Included in the refined and prioritised list of intervention components and the intervention prototype.

^b^ Consensus achieved (≥ 75% of participants selecting the same priority rating).

# Supplementary Table S11: Refined and prioritised list of components

| **Priority** | **Candidate component** | **Key design considerations** |
| --- | --- | --- |
| Must have | CDSS | Ensure the CDSS is clear and simple.  Base the CDSS on the 11-point threshold.  Provide the CDSS in more than one format, including:   - electronic format integrated with EHR systems and templates, which has automatic and transparent scoring, ideally auto-populates some of the variables (e.g. sex), and autosaves into patients’ notes - standalone website, which is ideally easily accessible via a link or standard search engine - electronic format completed by patients   Include one table with the predictor variables.  List the predictor variables in the following order: FDR, sex, smoking history and the joint pain variables from head to toe.  Provide the score for each predictor variable and the overall score output.  Automatically generate a summary output for sharing externally.  Ensure the decision tool has a memorable and relatively unique name. |
|  | Guidance on using the CDSS and undertaking the associated behaviours | Provide guidance through a flowchart alongside the decision tool (e.g., embedded in EHR systems and templates).  Provide brief guidance on using the CDSS and follow-up actions that covers:   - which patients to use / not use the CDSS with - how to assess the predictor variables (e.g. whether to assess current pain or pain over a specific timescale) - what actions to take based on the score output (including if/when the anti-CCP test should be repeated)   Provide guidance on patient education that covers:   - how to briefly discuss key points about / related to the CDSS with patients (e.g. how to arrange blood tests, how the test results will be used/followed up, implications of receiving a positive anti-CCP test result, safety-netting advice) - how to obtain information about rheumatology waiting times for providing to patients - how to help ensure patient discussions are helpful and reassuring for patients, for example by explaining:   - only a small proportion of patients who have an anti-CCP test will test positive   - only a small proportion of patients who test positive for anti-CCP will develop RA   - patients can proactively optimise their health through lifestyle changes   - early identification of RA improves patient outcomes   - being referred to rheumatology services has benefits   - effective management options are available for RA, and the prognosis is now much better than it used to be |
| Should have | Evidence supporting use of the CDSS | Ensure a brief easily accessible summary of the evidence is available alongside the CDSS, with the option of accessing more detailed evidence.  Provide evidence about:   - the IDEAS-PC prediction model properties (ideally including the negative predictive value and data on the proportion of people who develop RA with a year) - how the IDEAS-PC prediction model was developed - results of the health economic modelling (ideally incorporating whether patients would have had the anti-CCP blood test if the CDSS had not been used and whether they had any other blood tests) - the diagnostic test properties of the anti-CCP test - potential benefits and risks of identifying and people at risk of RA and potential referral, monitoring and intervention options |
|  | Patient education resources | Provide patient education resources that can be shared easily by clinicians and reviewed by patients independently.  Consider language barriers.  Consider providing some patient education when requesting the anti-CCP test.  Consider waiting until receiving the anti-CCP test results before providing more detailed education.  Include information about:   - the purpose of the anti-CCP test - what the test results mean, including potential implications such as affecting insurance policies - how the test results will be used / followed up - why patients may be referred rheumatology services (including the potential benefits of referral) - lifestyle advice - safety netting advice (including for if patients have ongoing or worsening symptoms) |
| Could have | Information covering key points about the CDSS | Provide information that highlights:   - who the intended users of the CDSS are. - potential benefits of using the CDSS, including for supporting clinical reasoning and improving patient outcomes - potential referral, monitoring and intervention options for people at risk of RA. - GPs were involved in developing the CDSS. - people who are anti-CCP negative may still develop RA. - the CDSS is just a decision aid and clinical judgement is needed to decide if the anti-CCP test is appropriate or a rheumatology referral is required (and provide evidence about how many patients who are anti-CCP negative develop RA if possible). - clinicians can show the CDSS to patients to support patient education and involve patients in their care. - providing appropriate education can help address patient anxiety. |

Anti-CCP, anti-cyclic citrullinated peptide; CDSS, clinical decision support system; EHR, electronic health record; FDR, first degree relative; IDEAS-PC, Improving iDEntification of rheumatoid ArthritiS in primary care; PAG, Project Advisory Group; RA, rheumatoid arthritis

# Supplementary Table S12: Phase 3 participant characteristics

| **Characteristic^1^** | **N (%)** |
| --- | --- |
| **Professional role** | |
| GP | 7 (58) |
| FCP with a physiotherapy background | 3 (25) |
| ANP | 2 (17) |
| **Years’ experience in current role** | |
| < 5 | 5 (42) |
| 5 < 15 | 4 (33) |
| ≥ 15 | 3 (25) |
| **Type(s) of organisation^2^** | |
| General practice | 9 (75) |
| GP confederation | 1 (8) |
| NHS community trust | 3 (25) |
| **Able to request the anti-CCP test** | |
| Yes directly | 11 (92) |
| Yes via another professional | 1 (11) |
| **Requested an anti-CCP blood test in the past 12 months** | |
| Yes | 12 (100) |
| **Integrated Care System area where workplace is located** | |
| Cheshire and Merseyside | 4 (33) |
| Dorset | 2 (17) |
| West Yorkshire | 6 (50) |
| **Recruitment approach** | |
| General practice | 4 (33) |
| Advertisement shared via professional network | 5 (42) |
| Not able to participate in Phase 2 but indicated they would like to participate in Phase 3 | 3 (25) |

^1^ Characteristics are only reported for roles in which clinicians undertake regular weekly clinics for a provider of NHS primary care services, which include assessing people with new-onset MSK symptoms without synovitis.

^2^ Participants could report more than one option.

Advanced Nurse Practitioner; anti-CCP, anti-cyclic citrullinated peptide; FCP, First Contact Practitioner; NHS, National Health Service.

# Supplementary Appendix S4: Could it Be RA? (COBRA) tool screenshots


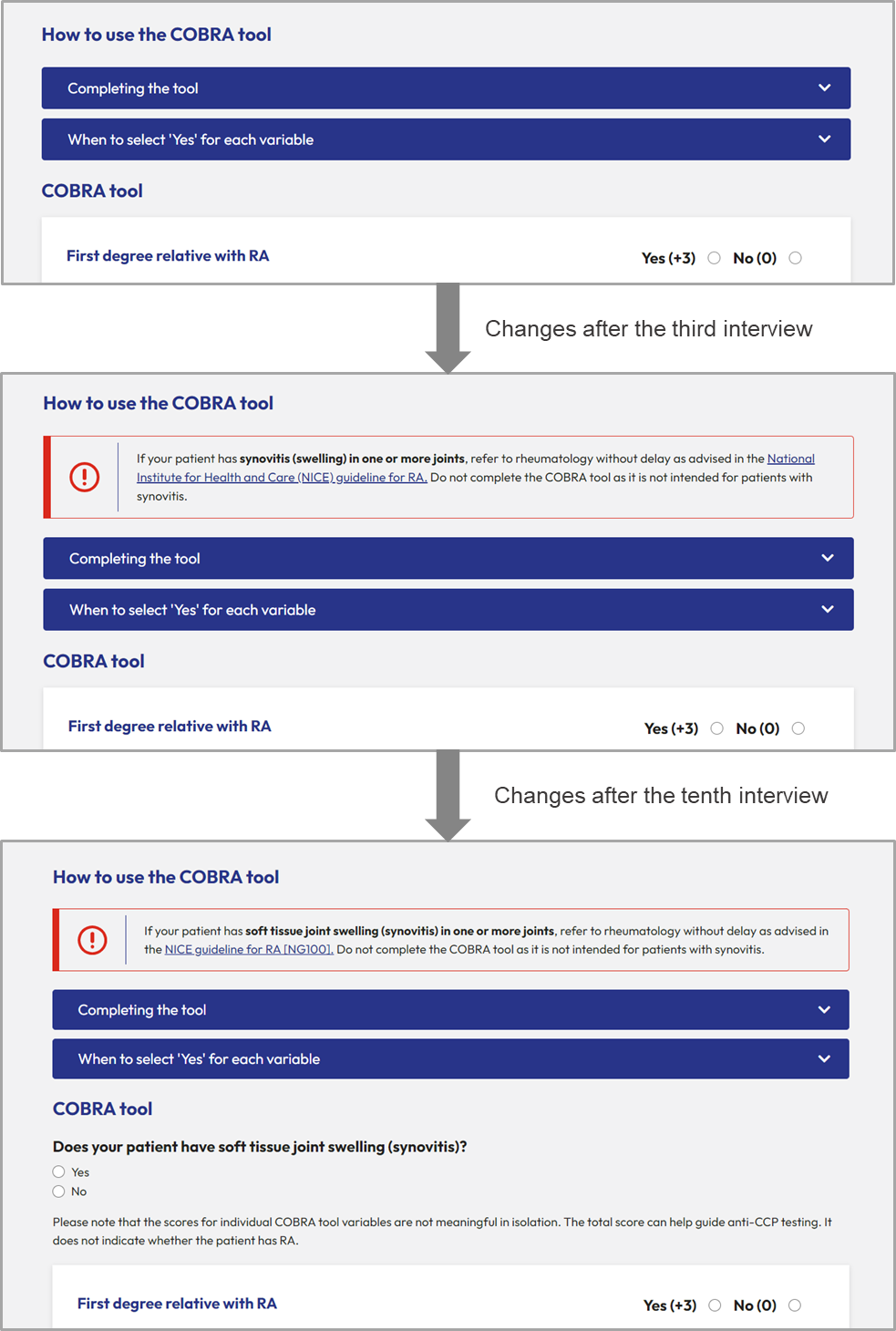


## Image description of the screenshots

Three screenshots of the COuld it Be RA? (COBRA) tool showing refinements to the instructions about when to use the COBRA tool.

### Initial version

The initial version includes a heading ‘How to use the COBRA tool’ and the following two dropdown boxes.

- Completing the tool.
- When to select ‘Yes’ for each variable.

Below the dropdown boxes is another heading ‘COBRA tool’. Below that heading is the score chart, which starts with ‘First degree relative with RA’ with radio buttons for ‘Yes (+3)’ and ‘No (0)’.

### Version after the third interview

The version after the third interview is the same as the initial version other than a red alert banner with an exclamation mark is included above the dropdown boxes. The alert banner states:

‘If your patient has synovitis (swelling) in one or more joints, refer to rheumatology without delay as advised in the National Institute for Health and Care (NICE) guideline for RA (hyperlink to guideline). Do not complete the COBRA tool as it is not intended for patients with synovitis.’

### Version after the tenth interview

The version after the tenth interview is the same as the version after the third interview other than a screening question is included below the ‘COBRA tool’ heading. The screening question states ‘Does your patient have soft tissue joint swelling (synovitis)?’ with ‘Yes’ and ‘No’ radio button options.

In addition, the following text is included above the score chart:

‘Please note that the scores for individual COBRA tool variables are not meaningful in isolation. The total score can help guide anti-CCP testing. It does not indicate whether the patient has RA.’

# Supplementary Appendix S5: Figure 1 image description

Flow chart summarising the three sequential project phases and Project Advisory Group meetings as follows. The intervention development domains are based on O’Cathain et al. (12).

## Phase 1

- Intervention development domain: Planning
- Design: Qualitative descriptive study
- Data collection: Semi-structured interviews
- Key output: List of candidate components

## Phase 2

- Intervention development domain: Designing and creating
- Design: Clinician consultation
- Data collection: Engagement workshops
- Key output: Intervention prototype

## Phase 3

- Intervention development domain: Refining
- Design: Think-aloud study
- Data collection: Concurrent think-aloud interviews
- Key output: Refined intervention

## Project Advisory Group meetings

Only Project Advisory Group actions that are directly focused on developing the intervention and logic model are listed.

- Meeting 1: Identify priorities for the intervention to feed into the list of candidate components in Phase 1.
- Meeting 2: Finalise the list of candidate components from Phase 1 and logic model v1.0 in preparation for Phase 2.
- Meetings 3–4: Finalise the intervention prototype and logic model v2.0 in preparation for Phase 3.
- Meeting 5: Finalise the refined intervention and logic model v3.0 so that the intervention is ready for feasibility testing.

# Supplementary Appendix S6: Text version of the COBRA tool flow

Assess patient with new-onset MSK symptoms.

If the patient **has** suspected persistent synovitis of undetermined cause, refer to rheumatology without delay.

If the patient does not have suspected persistent synovitis of undetermined cause, complete the COBRA tool.

If the COBRA tool score is less than 11, provide standard care, including safety netting.

If the COBRA tool score is more than or equal to 11, organise an anti-CCP test.

If the anti-CCP test result is negative, provide standard care, including safety netting.

If the anti-CCP test result is positive, seek rheumatology guidance.

See the [NICE RA guideline](https://www.nice.org.uk/guidance/ng100/chapter/Recommendations) for more guidance on referring patients with suspected persistent synovitis of undetermined cause.

# Supplementary Appendix S7: Figure 3 image description

Logic model summarising how the Could it Be RA? (COBRA) tool is intended to improve clinical and economic outcomes as follows.

## Problem to address

The anti-cyclic citrullinated peptide (anti-CCP) prediction model could support clinicians to identify people at risk of rheumatoid arthritis (RA) in primary care by targeted anti-CCP testing. However, implementing the prediction model in primary care is likely to be challenging.

## Intervention components

- Clear and simple clinical decision support system (CDSS) (score chart based on the anti-CCP prediction model, which predicts whether a patient is likely to test positive for anti-CCP).
- Brief guidance on using the CDSS and the associated behaviours listed below.
- Information covering key points about the CDSS.
- Evidence supporting use of the CDSS.
- Patient education resources related to the CDSS.

## Intended change mechanisms

### Intervention processes linked to the Capability Opportunity Motivation model of behaviour (COM-B) (8)

- Enable clinicians to access and use the CDSS quickly and easily (increase physical opportunity).
- Equip clinicians with the knowledge, psychological skills, and confidence to use the CDSS and undertake the associated behaviours in the primary care context (increase psychological capability, physical opportunity, and reflective motivation).
- Influence interpersonal factors and cultural norms related to using the CDSS and undertaking the associated behaviours (increase social opportunity).
- Address clinicians’ concerns and increase their beliefs about the benefits of using the CDSS and undertaking the associated behaviours (increase reflective motivation).
- Enable clinicians to easily access patient education resources and address concerns related to increasing patients’ anxiety (increase physical opportunity, social opportunity and reflective motivation).

### Intended behaviours of primary care clinicians

- Use the CDSS with appropriate patients.
- Organise for patients to have an anti-CCP blood test when supported by the CDSS.
- Seek rheumatology guidance for patients with a positive anti-CCP test.
- Have appropriate discussions about anything related to RA, anti-CCP testing or rheumatology referrals with patients.

### Mediators

- People at risk of RA monitored in primary care or rheumatology services if indicated.
- People at risk of RA aware of signs / symptoms suggestive of RA onset and how to seek help if needed.

## Intended clinical and health economic outcomes

- Earlier identification of RA (including identification at the onset of overt signs and symptoms, and at the onset of subclinical inflammation identified through imaging) and more rapid treatment initiation.
- Delaying or preventing the onset of RA.
- Prevention / minimisation of joint damage and disability and improved health-related quality of life.
- Cost savings through lower healthcare resource utilisation.

## Potential unintended consequences

- Increased anxiety and early disability among people at risk of RA (which clinicians having appropriate discussions with patients is hypothesised to help prevent).
- Increased workload for primary care and rheumatology services (which prevention / minimisation of joint damage and disability and improved health-related quality of life is hypothesised to help prevent).

## Contextual factors

- Primary care staffing and resources, including clinicians’ professional backgrounds and experience, appointment delivery modes, appointment durations, other time constraints, and Information Technology (IT) systems.
- Primary care caseloads, including the volume of patients the CDSS could be used with and who would require follow up, and their health literacy and sociodemographic characteristics.
- Systems for organising anti-CCP testing and rheumatology referrals, including which clinicians can request the anti-CCP test and receive the results, which clinicians can refer patients to rheumatology services, how easy / difficult the referral forms are to access and use, what referral pathways are in place, and whether clinicians can directly book patient appointments.
- Multidisciplinary team relationships and cultural factors, including whether General Practitioners trust First Contact Practitioners / Advanced Practitioners to act autonomously, clinicians’ cultural beliefs about the anti-CCP test and rheumatology referrals, and the relationship between the primary care and rheumatology teams.
- Relevant policies and guidelines (e.g. from the National Institute for Health and Care Excellence (NICE), NHS England, and the British Society for Rheumatology) and financial considerations (e.g. funding for the CDSS).

# References

1. Anderson AM, Richards S, Flurey C, Siddle HJ. Identifying people at risk of rheumatoid arthritis in primary care: qualitative study. Br J Gen Pract. 2025:BJGP.2024.0590.

2. Braun V, Clarke V. Successful qualitative research: a practical guide for beginners. London: SAGE Publications Ltd; 2013.

3. Heyhoe J, Reynolds C, Bec R, Wolstenholme D, Grindell C, Louch G, Lawton R. The Shared Safety Net Action Plan (SSNAP): a co-designed intervention to reduce delays in cancer diagnosis. Br J Gen Pract. 2022;72(721):e581-e91.

4. Turner J, Martin G, Hudson N, Shaw L, Huddlestone L, Weis C, et al. Using Normalisation Process Theory (NPT) to develop an intervention to improve referral and uptake rates for self-management education for patients with type 2 diabetes in UK primary care. BMC Health Serv Res. 2022;22(1):1206.

5. Nielsen J. Estimating the number of subjects needed for a thinking aloud test. Int J Hum Comput. 1994;41(3):385-97.

6. Bradbury K, Morton K, Band R, van Woezik A, Grist R, McManus RJ, et al. Using the Person-Based Approach to optimise a digital intervention for the management of hypertension. PLoS One. 2018;13(5):e0196868.

7. Bishop FL, Greville-Harris M, Bostock J, Din A, Graham CA, Lewith G, et al. Using psychological theory and qualitative methods to develop a new evidence-based website about acupuncture for back pain. Europ J Integr Med. 2016;8(4):384-93.

8. Michie S, van Stralen MM, West R. The behaviour change wheel: A new method for characterising and designing behaviour change interventions. Implement Sci. 2011;6(1):42.

9. Michie S, Atkins L, West R. The Behaviour Change Wheel: A Guide to Designing Interventions. London, UK: Silverback Publishing; 2014.

10. Michie S, Richardson M, Johnston M, Abraham C, Francis J, Hardeman W, et al. The behavior change technique taxonomy (v1) of 93 hierarchically clustered techniques: building an international consensus for the reporting of behavior change interventions. Ann Behav Med. 2013;46(1):81-95.

11. Garcia-Montoya L, Nam JL, Duquenne L, Villota-Eraso C, Di Matteo A, Hartley C, et al. Prioritising referrals of individuals at-risk of RA: guidance based on results of a 10-year national primary care observational study. Arthritis Res Ther. 2022;24(1):26.

12. O’Cathain A, Croot L, Sworn K, Duncan E, Rousseau N, Turner K, et al. Taxonomy of approaches to developing interventions to improve health: a systematic methods overview. Pilot Feasibility Stud. 2019;5(1):41.

1. Kuhn J. Decrypting the MoSCoW analysis. DITY weekly newsletter, itSM Solutions. 2009;5(44).

   Bradbury K, Watts S, Arden-Close E, Yardley L, Lewith G. Developing Digital Interventions: A Methodological Guide. Evid Based Complement Alternat Med. 2014;2014:561320. [↑](#footnote-ref-1)
2. Michie S, Atkins L, West R. The Behaviour Change Wheel: A Guide to Designing Interventions. London, UK: Silverback Publishing; 2014. [↑](#footnote-ref-2)
